# α-Amanitin Restrains Cancer Relapse from Drug-Tolerant Cell Subpopulations via *TAF15*

**DOI:** 10.1038/srep25895

**Published:** 2016-05-16

**Authors:** Kohei Kume, Miyuki Ikeda, Sawako Miura, Kohei Ito, Kei A. Sato, Yukimi Ohmori, Fumitaka Endo, Hirokatsu Katagiri, Kaoru Ishida, Chie Ito, Takeshi Iwaya, Satoshi S. Nishizuka

**Affiliations:** 1 Molecular Therapeutics Laboratory, Department of Surgery, Iwate Medical University School of Medicine, Morioka, Iwate 020-8505, Japan; 2 MIAST (Medical Innovation by Advanced Science and Technology) project, Iwate Medical University School of Medicine, Morioka, Iwate 020-8505, Japan; 3 Institute of Biomedical Science, Iwate Medical University, Yahaba, Iwate 028-3694, Japan

## Abstract

Cancer relapse occurs with substantial frequency even after treatment with curative intent. Here we studied drug-tolerant colonies (DTCs), which are subpopulations of cancer cells that survive in the presence of drugs. Proteomic characterization of DTCs identified stemness- and epithelial-dominant subpopulations, but functional screening suggested that DTC formation was regulated at the transcriptional level independent from protein expression patterns. We consistently found that α-amanitin, an RNA polymerase II (RNAPII) inhibitor, effectively inhibited DTCs by suppressing *TAF15* expression, which binds to RNA to modulate transcription and RNA processing. Sequential administration of α-amanitin and cisplatin extended overall survival in a cancer-relapse mouse model, namely *peritonitis carcinomatosa*. Therefore, post-treatment cancer relapse may occur through non-distinct subpopulations and may be effectively prevented by α-amanitin to disrupt transcriptional machinery, including *TAF15*.

Cancer relapse after chemotherapy is a biological phenomenon wherein some drug-tolerant cells in heterogeneous cell populations robustly survive and propagate under conditions of stress. In the case of advanced gastrointestinal tumors, 50–90% of cancer patients die due to disease relapse despite having undergone apparently “curative” resection, which is defined by a status where no detectable cancer cells remain in the patient’s body^1^, followed by adjuvant chemotherapy^2^. However, relapse can occur even during adjuvant chemotherapy, suggesting that small populations of cancer cells can survive chemotherapy and promote local tumor growth, lymph node/hematological metastases, and pleural/peritoneum disseminations. Recent studies have demonstrated that several decades are needed for some tumors to grow to lethal levels as a result of the accumulation of genetic alterations^3^,^4^. Most relapses occur within five years after curative treatment of gastrointestinal cancer patients, which is a much shorter duration than the estimated time needed for de novo cancers to acquire highly malignant phenotypes^5^. Therefore, rather than having an underlying acquired genetic cause, we hypothesized that the mechanism underlying relapse is based on functional changes, including chromatin structure alterations, selective gene expression, or population selection, which are associated with drug resistant and highly malignant phenotypes^6^,^7^,^8^. As such, identifying target molecules that can restrain cell division and subsequent propagation following chemotherapy is important for improving patient outcomes. However, the identification of such targets may be difficult due to the variety of targets that can mediate different survival mechanisms of heterogeneous populations depending on differences in drug and cell types^9^. Therefore, agents that are capable of restraining cancer relapse after chemotherapy should possess inhibitory mechanism(s) that can affect several fundamental cancer cell functions and cross the actions of many different drugs.

To elucidate the mechanisms of cancer relapse, we used drug-tolerant colonies (DTCs), which are subpopulations that survive in the presence of anticancer drugs. Since profiling individual DTCs at a molecular level may reveal functional subgroups that are associated with drug-tolerant phenotypes, we first developed colony lysate arrays (CoLAs) that allowed for a comparison of a few thousand individual colony-driven lysates with a set of functionally relevant protein levels. We then examined different molecular mechanisms at the level of chromatin modification, transcription, and translation. Based on the proteomic profiles and functional level examinations, transcriptional regulation appeared to be a key underlying mechanism for DTC formation. We further investigated the impact of RNA polymerase II (RNAPII) inhibition on DTC survival as well as the prevention of peritonitis carcinomatosa (PC), which is one of the most devastating terminal cancer statuses, when RNAPII inhibition and cisplatin (CIS) are sequentially administered.

Here we report that the RNAPII inhibitor α-amanitin (α-AMA) in combination with CIS plays a significant role in the suppression of PC. We demonstrate the potential clinical utility of an RNAPII inhibitor for malignancies having a high probability of developing PC that may result from the highly heterogeneous features of DTCs.

## Results

### Distinct biological properties of cancer cell sheets and colonies

**Figure 1:**
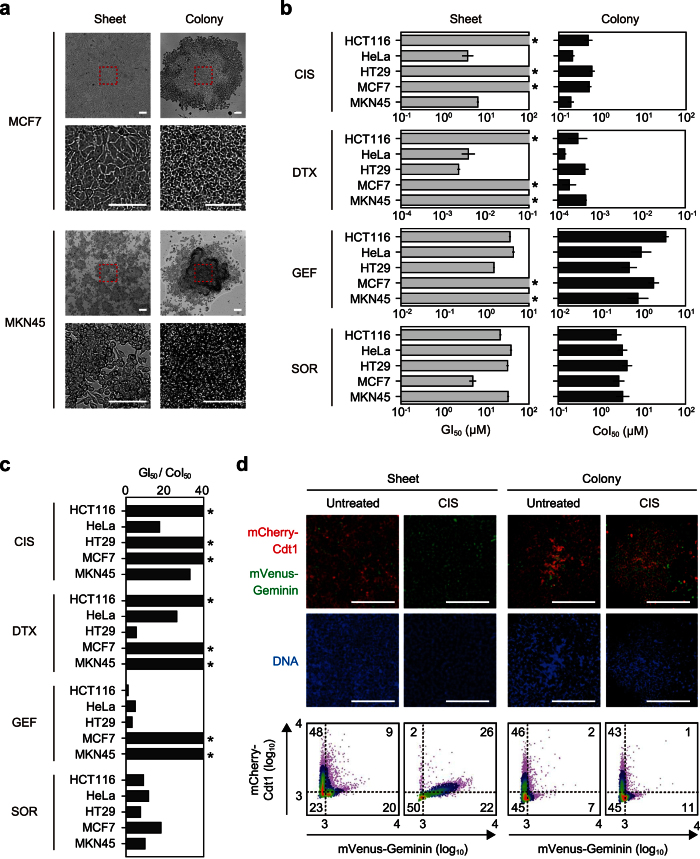
Distinct biological and properties of cancer cell sheets and colonies. (**a**) Representative images of sheet and colony morphology of the indicated cell lines. Scale bars = 100 μm. (**b**) Drug sensitivities of cells in sheets and colonies. GI_50_ and CoI_50_ concentrations for each cell line were determined by a growth suppression assay and colony formation assay, respectively. Asterisks indicate no inhibition. All experiments were performed in triplicate. Error bars represent s.e.m. (**c**) GI_50_/CoI_50_ ratios of each drug were calculated using data in Fig. 1b. Asterisks indicate the ratio is more than 40-fold. (**d**) Cell cycle state of HeLa.S-Fucci2 cells in sheets and colonies. Top panels show fluorescent images of mCherry-hCdt1 (red) and mVenus-hGeminin (green) expression. Hoechst 33342 (blue) was used to stain nuclear DNA. Scale bars = 100 μm. Corresponding density scatter plots with the threshold of red, green, yellow and no color cells are shown (bottom panels). Numbers indicate percentages of each fraction.

Growth suppression assays of exponentially growing cells reflect the capacity of a drug to reduce or stabilize the size of solid tumors, whereas colony formation assays mimic relapse from a minimum number of cancer cells as a result of drug-tolerant tumor initiating single cells^10^. We hypothesized that the biological properties of cells during exponential growth and colony formation may differ in terms of their proliferative potential. For instance, morphological examination indicated that cells in the exponential growth phase spread two-dimensionally in a flask (i.e., sheet), whereas colony-forming cells showed dense, small, and vertical growth (Fig. 1a; Supplementary Fig. S1). Drug concentrations required to suppress colony formation of five cancer cell lines by 50% (CoI_50_) were 1–3 orders of magnitude lower than those needed for growth suppression (GI_50_) for the conventional chemotherapeutic agents CIS and docetaxel (DTX). Moreover, the CoI_50_ for colony formation of molecular targeting drugs gefitinib (GEF) and sorafenib (SOR) was 10-fold less than that of the corresponding GI_50_ for cell growth (Fig. 1b,c; Supplementary Fig. S2). A fluorescent ubiquitination-based cell cycle indicator (Fucci)^11^ system was used to assess differences in cell cycle distribution between sheets and colonies. In sheets, typical fractions undergoing cell cycle onset were observed (Fig. 1d; Supplementary Fig. S3). In contrast, the cell cycle distribution of the cells in colonies was not detectably altered by CIS. These results suggest that sheets and colonies represent different states for cancer cells, particularly in terms of drug response.

### Functional heterogeneity of cancer cell colonies

**Figure 2:**
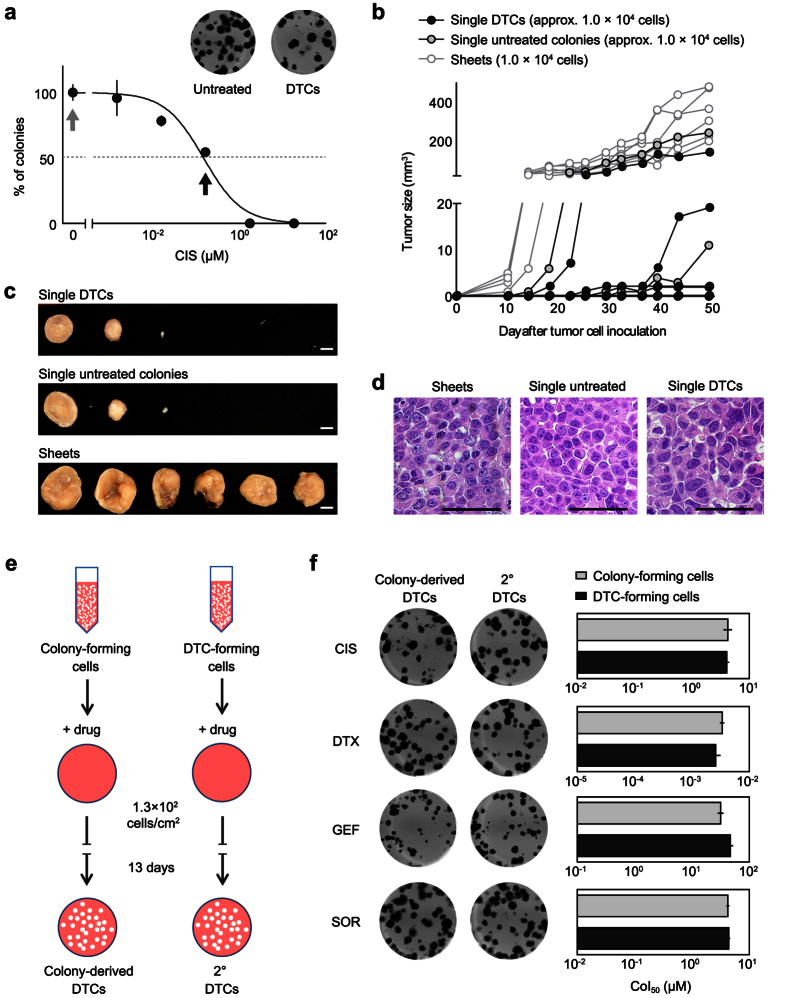
Functional heterogeneity of cancer cell colonies. (**a**) MKN45-derived untreated colonies and DTCs. CIS concentrations for the emergence of the colonies in a dose-response curve are shown. Untreated colonies were obtained in absence of CIS (light gray arrow) and DTCs were obtained after treatment with CIS at CoI_50_ (black arrow). Each experiment was performed in triplicate. Error bars represent s.e.m. (**b**) Tumor-forming capacity of individual DTCs and untreated colonies derived from MKN45. A single, one mm-diameter DTC and untreated colony (containing approximately 1.0 × 10^4^ cells) were randomly picked and individually inoculated subcutaneously (*n* = 6). Sheet-derived 1.0 × 10^4^ cells were used as a control. Tumor size was measured twice weekly. (**c**) Tumors were removed at 7 weeks after inoculation. Scale bar = 5 mm. (**d**) Pathological examination of sheet-, untreated colony-, and DTC-derived tumors with H & E staining. Scale bars = 500 μm. (**e**) An illustration for comparing drug sensitivity between colony-forming cells and DTC-forming cells. Untreated colonies and DTCs were diluted into single cell suspensions and re-seeded into wells with a CoI_50_ concentration of each drug. (**f**) Representative wells with colony-derived DTCs and secondary DTCs (left), and drug sensitivities of untreated colony- and DTC-forming cells (right). CoI_50_ concentrations were determined based on dose-response curves for the number of colonies. Each experiment was performed in triplicate. Error bars represent s.e.m.

DTCs are defined as colonies that can survive in the presence of anticancer drugs at respective CoI_50_ concentrations (Fig. 2a; Supplementary Fig. S4). Even in the face of drug treatment, resting or slowly proliferating cells can endure and reinitiate tumor growth^8^. To evaluate whether DTCs possess higher tumorigenicity than both untreated colonies and sheets, we subcutaneously inoculated single DTCs, single untreated colonies, or an equal number (1.0 × 10^4^) of sheet-derived cells into nude mice. MKN45 cells were used for inoculation because of the highest colony-forming ability among all cell lines tested (Supplementary Fig. S5). Cells from sheets showed continuous growth in all replicates, whereas only 50% (3/6) of recognizable tumors formed after the inoculation of DTCs and untreated colonies (Fig. 2b,c). The size variations of tumors derived from single DTCs and single untreated colonies suggest that chemotherapy does not necessarily induce or enrich “drug-tolerant” clones^8^,^12^. Regardless of whether the cells used for xenografting were derived from sheets, colonies, or DTCs, there all have morphological similarities based on histology (Fig. 2d). The variable degree of tumor growth from both DTCs and untreated colonies *in vivo* may reflect non-deterministic mechanisms relevant to tumor growth rates.

We next investigated whether DTCs have a reversible phenotype based on the finding that a chromatin-mediated mechanism regulates gene expression in drug-tolerant cancer cells.6 If a reversible mechanism plays a role in forming DTCs, then re-formation of DTCs from single-cell suspensions may “reboot” regulatory mechanisms responsible for DTC formation (Fig. 2e). The numbers of secondary DTCs were similar from re-disseminated untreated colonies (Fig. 2f, left). The CoI50 values after re-dissemination of DTCs were not higher than those of untreated colonies (Fig. 2f, right; Supplementary Fig. S6). These results suggest that the drug-tolerant properties of DTCs were lost during the processes involved in single cell suspension, re-dissemination, and re-formation of colonies due to the same reversible mechanisms.

### Two major subgroups of DTCs in terms of proteomic profile

**Figure 3:**
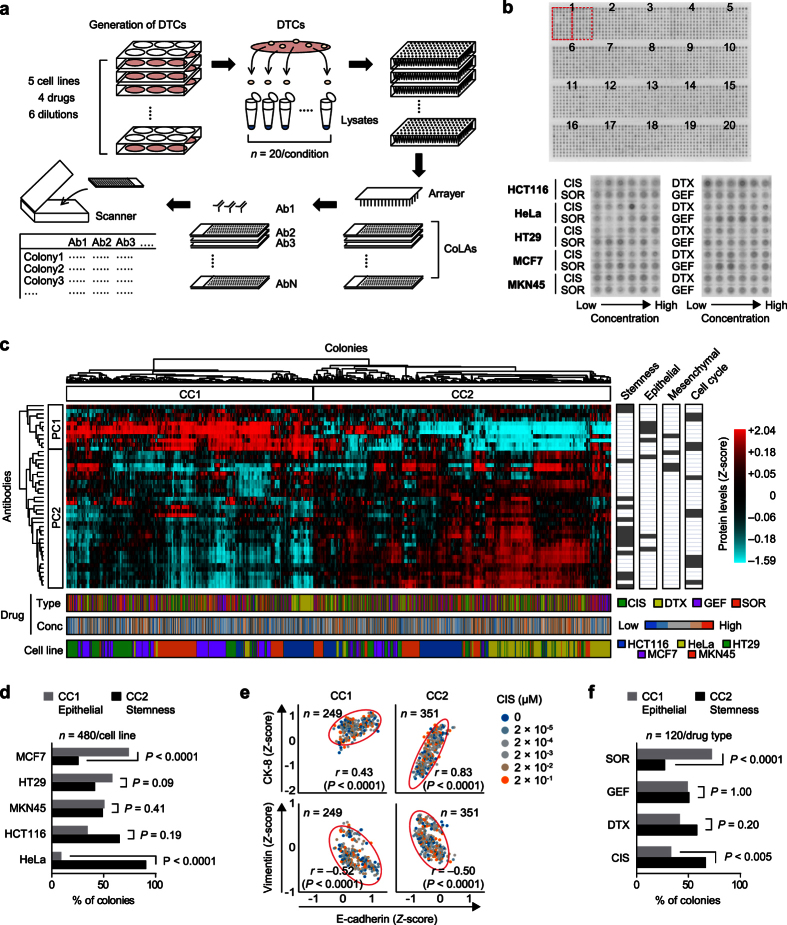
Protein levels in individual DTCs. (**a**) Schematic overview of CoLA analysis. A total of 2400 colonies including both DTCs and untreated colonies were generated by a colony formation assay. Colonies were then individually isolated, lysed and arranged in 384-well plates. The lysates were spotted onto a glass slide embedded with a nitrocellulose membrane to produce a CoLA. Each CoLA was then stained with a specific primary antibody and scanned to quantify spot intensities. (**b**) The CoLA platform. Spots were arranged into five columns and four rows for 20 sets of biological replicates (top panel, colloidal gold stain). Each set contains colony lysates from different drug treatment conditions of five cell lines indicated in red boxes (bottom panel). (**c**) Protein levels of 2400 individual colony lysates were classified by two-way hierarchical clustering. Drug types, concentrations and cell lines are indicated at the bottom. Functional categories of proteins include stemness, epithelial, mesenchymal, and cell cycle markers as indicated on the right. Two major clusters were evident in both the colony axis (CC1 and CC2) and protein axis (PC1 and PC2). Values were normalized by colloidal gold-stained total protein and *Z*-score transformation was used for analysis. (**d**) The distribution of cell line in CC1 and CC2. The two-sided *P* values were obtained with Fisher’s exact test. (**e**) Correlation of epithelial (E-Cadherin and CK-8) and mesenchymal markers (vimentin) in individual colonies including both DTCs and untreated colonies from CIS condition. Scatter plots of E-Cadherin vs. CK-8 (top panels) or vimentin (bottom panels) are shown with Pearson’s correlation coefficient (*r*) used to assess the correlations. (**f**) The distribution of drug type in CC1 and CC2 in MKN45 DTCs. The two-sided *P* values were obtained with Fisher’s exact test.

To assess changes in the levels of protein markers in DTCs, we performed CoLA assays, which involve a modified reverse-phase protein microarray (RPPA) that uses specific antibodies to detect various kinds of proteins in a quantitative manner^13^,^14^. We first collected a total of 2400 colonies from five cell lines (HCT116, HT29, HeLa, MCF7, and MKN45) that were exposed to four different kinds of drugs (CIS, DTX, GEF, and SOR) at five different concentrations, as well as from those without drugs (Fig. 3a; Supplementary Table S1). Cells from these 2400 colonies were then lysed and printed onto nitrocellulose attached to a glass slide (Fig. 3a,b). Quantitative protein measurement was performed on the CoLAs using 44 specific antibodies to assess stemness, pluripotency, and epithelial and mesenchymal markers (Supplementary Table S2). Two-way hierarchical clustering identified two major colony clusters (CC1 and CC2) and two major protein clusters (PC1 and PC2) (Fig. 3c; Supplementary Fig. S7). CC1 showed high and low levels of epithelial and stemness marker proteins, respectively, whereas CC2 exhibited a reciprocal phenotype. MCF7 and HeLa were significantly enriched in CC1 or CC2 (P < 0.001), respectively, whereas HCT116, HT29, and MKN45 were spread in both clusters (Fig. 3d). Interestingly, the epithelial/non-epithelial relationship indicated that E-cadherin was positively and negatively correlated with CK-8 and vimentin, respectively, in DTCs independent from all drug concentrations (Fig. 3e; Supplementary Fig. S8). These observations suggest that the two distinct major subgroups of DTCs from any drug condition either existed prior to the drug treatment or were stochastically induced.

### Initiation and establishment of DTCs

**Figure 4:**
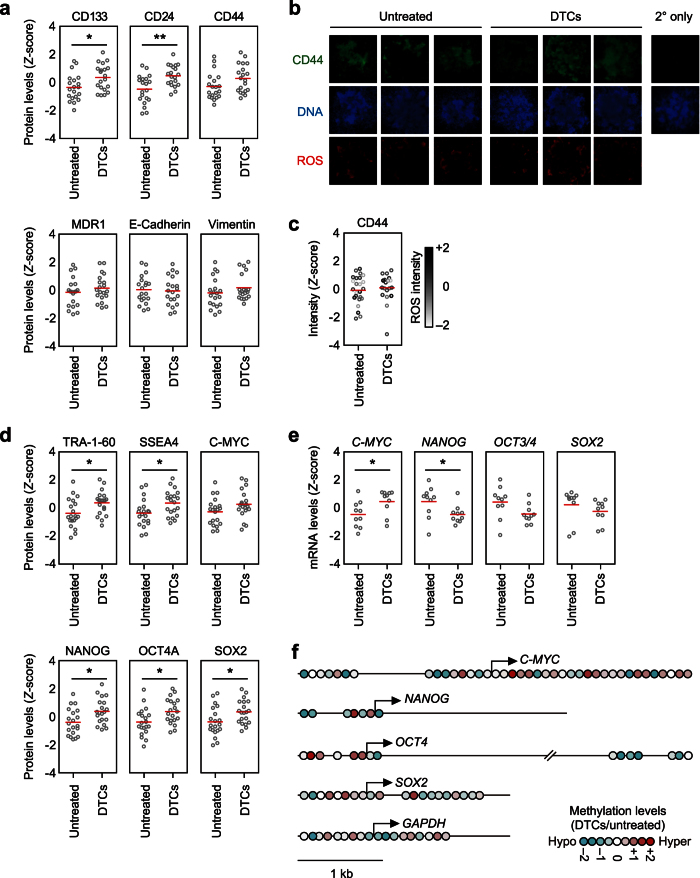
Association between DTC formation and transcriptional regulation. (**a**) CoLA analysis of the indicated cell surface markers in individual untreated colonies and DTCs of MKN45. MKN45 DTCs emerged in the presence of 0.2 μM CIS. Red lines indicate the mean values of individual colonies for each condition. *P < 0.05, **P < 0.01, Student’s *t* test. (**b**) Validation of CoLA data using fluorescence immunocytochemistry. Representative colonies that stained with anti-CD44 antibodies (green), ROS indicator (red), and Hoechst 33342 for DNA (blue) are shown. (**c**) Corresponding dot plots show the fluorescence intensity of indicated proteins in individual untreated colonies and DTCs. Black to white gradient indicates ROS intensity of individual colonies. (**d**) CoLA analysis of indicated pluripotency-associated proteins. *P < 0.05, Student’s *t* test. (**e**) The abundance of indicated mRNAs was quantified using quantitative (q)RT-PCR (normalized by *GAPDH* mRNA levels). Red lines indicate the mean values of individual colonies for each condition. *P < 0.05, Student’s *t* test. (**f**) Relative methylation levels of CpG regions located at the transcription start sites of pluripotency-inducing genes. *GAPDH* is shown as a housekeeping gene.

The hierarchical clustering from the CoLA assay revealed that MKN45 colonies had the most plastic phenotype. Since treatment of MKN45 with CIS was associated with an epithelial-low/stemness-high phenotype, the epithelial-low/stemness-high phenotype seemed to be a marker for the DTCs (Fig. 3f). We next investigated the difference in individual protein levels of putative markers for the cancer stem cells (CSC) and the epithelial-mesenchymal transition (EMT) in both CIS-treated and untreated colonies of MKN45. Although levels of several markers, including CD133 and CD24^15^,^16^, were significantly higher in MKN45 DTCs, none of these markers were exclusively expressed in the DTCs (Fig. 4a), which is consistent with the wide variation of tumorigenicity in individual DTCs (Fig. 2b,c). Moreover, fluorescent immunocytochemistry of CD44 in DTCs and untreated colonies was heterogeneous in both groups (Fig. 4b,c). Although high levels of CD44 have been shown to antagonize reactive oxygen species (ROS)^17^, ROS was indeed observed in both DTCs and untreated colonies. Moreover, CD44 intensity showed no significant correlation with the ROS levels in individual colonies (Fig. 4b,c).

*S*ince changes in levels of the putative CSC and EMT markers did not seem to be directly related to a drug-tolerant phenotype, we next focused on pluripotency-associated proteins. The levels of the pluripotency-associated proteins, including SSEA4, TRA-1-60, NANOG, OCT4A, and SOX2^18^,^19^, were consistently higher in MKN45 DTCs than those of untreated colonies (Fig. 4d). In contrast to the levels of pluripotency-associated proteins, the mRNA level of pluripotency-inducing transcription factors was downregulated in MKN45 DTCs relative to untreated colonies (Fig. 4e). The methylation status of upstream regions of transcription start sites for pluripotency-inducing genes was in good agreement with their gene expression levels (Fig. 4f). Interestingly, hierarchical clustering in both proteins and transcripts identified two major clusters that discriminated DTCs from untreated colonies (Supplementary Fig. S9). The gene expression of pluripotent-inducing factors may be induced and reduced immediately once cells are exposed to drugs, and the proteins remain present longer to establish the drug-tolerant phenotype. This observation may reflect the time lag between transcriptional control and protein turnover in the context of controlling stemness properties in DTCs^20^. Taken together, these observations led us to hypothesize that the stochastic emergence of DTCs may be initiated by transcriptional regulation and subsequently established by protein expression.

### High efficacy of RNAPII inhibition on DTC formation

**Figure 5:**
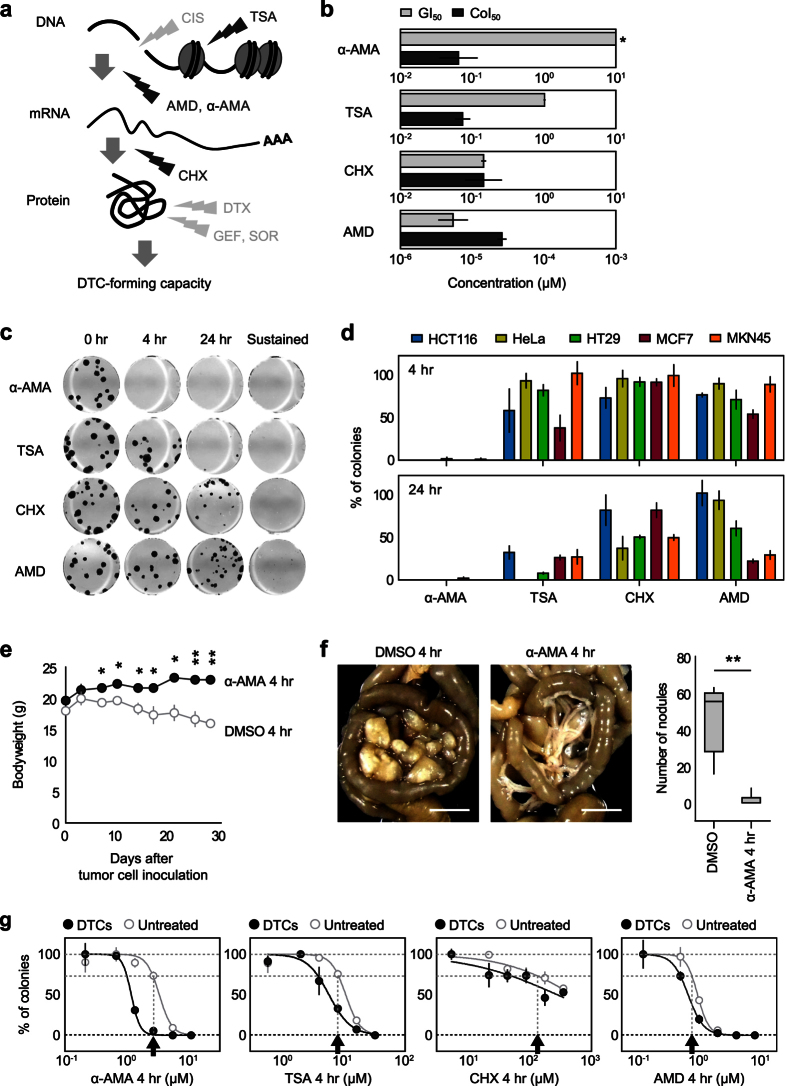
RNAPII-dependent colony-forming capacity. (**a**) Schematic overview of molecular targeted fractions in the context of gene expression and protein synthetic processes. (**b**) Inhibitory compound screening based on GI_50_ and CoI_50_. GI_50_ and CoI_50_ values of trichostatin A (TSA), actinomycin D (AMD), α-amanitin (α-AMA) and cycloheximide (CHX) are shown. All experiments were performed in triplicate. Error bars represent s.e.m. An asterisk indicates no inhibition. (**c**) Inhibition of colony formation by temporal treatment (0, 4 and 24 hours) with each compound. Black arrows indicate a concentration that is 50-fold greater than the CoI_50_ value for each compound, which is the concentration used for colony formation assay with temporal drug treatment. (**d**) Validating the effect of α-AMA treatment on colony formation in five cancer cell lines. Cells were temporally (4 or 24 hours) treated with each compound prior to cell seeding. Colony numbers relative to those with no treatment are shown as a percentage. (**e**) Body weights of nude mice after peritoneal injection of MKN45 cells treated with DMSO or α-AMA for 4 hours. Each group of experiments was performed in replicates (*n* = 3). Error bars represent s.e.m. *P < 0.05, **P < 0.01, Student’s *t* test. (**f**) Mesentery with disseminated and mature peritoneal nodules in DMSO or α-AMA treated groups (left). The number of nodules at 28 days after peritoneal injection of MKN45 cells (right). Each group of experiments was performed in a set of six biological replicates. Error bars represent s.e.m. **P < 0.01, Student’s *t* test. (**g**) MKN45 cells were temporally (4 hr) treated with two-fold serial dilution of α-AMA, TSA, CHX, and AMD prior to colony formation in the presence of 0.2 μM CIS. Black arrows indicate CoI_25_ value of CIS-untreated colonies for each compound.

To confirm the transcriptional steps that predominantly affect the emergence of DTCs, we screened a set of four compounds that affect steps, including chromatin formation, transcription, or protein synthesis (Fig. 5a). Trichostatin A (TSA) is an antifungal antibiotic that inhibits class I/II histone deacetylases (HDACs)^21^. Actinomycin D (AMD) is a cyclic polypeptide-containing antibiotic that has clinical applications for treating multiple types of sarcoma by acting as an inhibitor of RNAPI (at 0.05 μg/ml), RNAPII (at 0.5 μg/ml), and RNAPIII (at 5 μg/ml)^22^. α-AMA is a mushroom toxin that inhibits RNAPII^22^,^23^. In addition, cycloheximide (CHX) is an antifungal antibiotic that inhibits translational peptide chain elongation^24^.

We first examined colony-specific inhibition of these compounds by comparing GI50 and CoI50 values. TSA and α-AMA exhibited more than 10-fold lower CoI50 than GI50 (Fig. 5b). We next determined the CoI50 concentration for each inhibitor and 50-fold higher concentration of the respective CoI50 values were used for 4 and 24-hour exposure of inhibitors in a colony formation assay. TSA clearly suppressed colony formation after 24-hour exposure (Fig. 5c). A complete suppression of colony formation by α-AMA was observed after 4- and 24-hour exposure (Fig. 5c). On the other hand, the less-specific RNAP inhibitor AMD showed no significant colony suppression after 4- and 24-hour exposure, suggesting that the selective inhibition of RNAPII plays a significant role in the inhibitory effect of colony formation (Fig. 5c). CHX produced a slight suppression of colony formation, but the effect of CHX-induced inhibition of protein synthesis reductions in colony formation seemed to be milder than that of epigenetic regulation and mRNA synthesis (Fig. 5c). We also confirmed that both TSA and α-AMA demonstrated remarkable colony suppression following 24-hour exposure, while exposure to α-AMA for 4 hours was sufficient for a nearly complete colony suppression effect for all cell lines tested (Fig. 5d). Although chromatin modification may be one of the major causes behind alterations in gene expression during colony formation^25^, these findings suggest that RNAPII-dependent inhibition of early-phase mRNA synthesis is sufficient to produce a nearly complete suppression of colony formation. The significance of mRNA synthesis during colony formation was also demonstrated in a colony formation assay-based screening with a compound library showing that RNA polymerase inhibition was ranked among the top 2.5% categories of 160 mechanism of actions (Supplementary Fig. S10).

To examine whether colony suppression can be reproduced *in vivo*, we next observed nude mice after peritoneal injection of 1.0 × 10^6^ MKN45 cells that had been treated with α-AMA for 4 hours. Mice receiving a peritoneal injection of MKN45 cells showed a constant decrease in body weight, while those mice injected with α-AMA-treated cells maintained their body weight, likely due to reduced alimentary tract functional failure (Fig. 5e). At 28 days after peritoneal injection of cancer cells, the number of peritoneal nodules was significantly lower in mice that received α-AMA-treated cancer cells relative to those injected with untreated MKN45 cells (Fig. 5f). A subsequent colony formation assay showed that α-AMA has the most potent activity in DTC suppression among the four compounds (Fig. 5g).

### RNAPII-dependent *TAF15*-mediated drug tolerance

**Figure 6:**
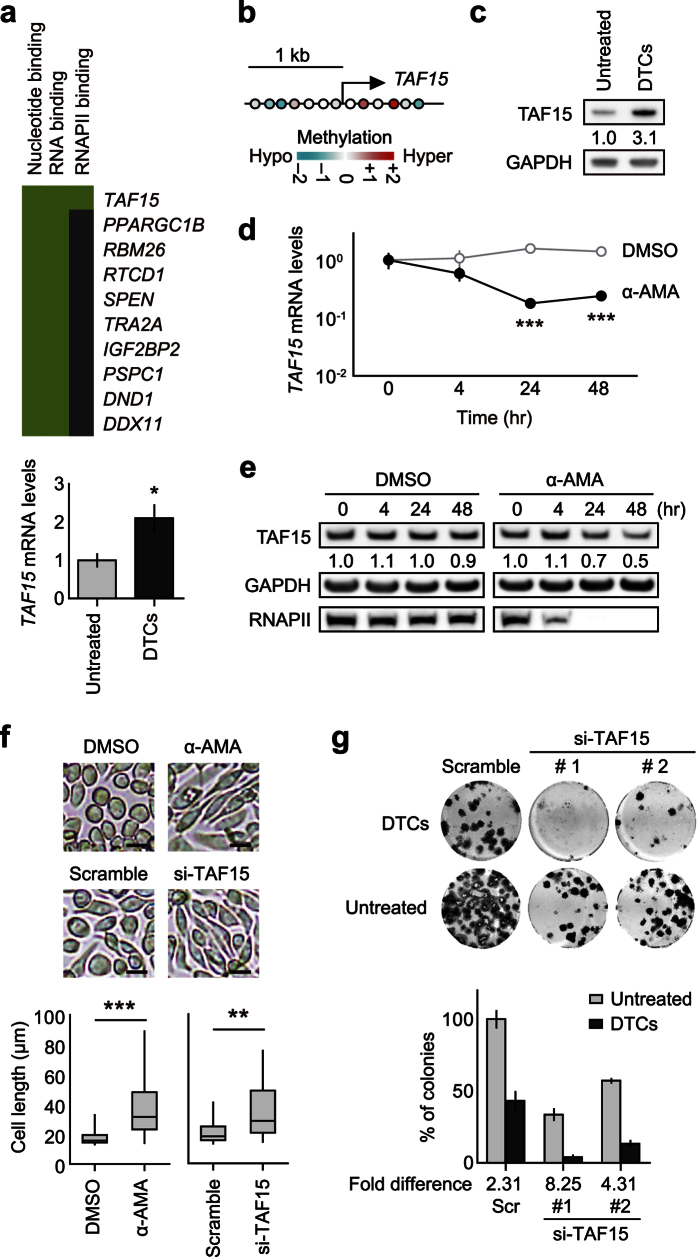
Identification of *TAF15* as a mediator of RNAPII activity against DTC formation. (**a**) RNA-binding protein-coding genes. The green area shows corresponding gene-term association previously reported (top panel). The expression level of *TAF15* mRNAs was quantified using qRT-PCR (relative to *GAPDH* mRNA levels, bottom panel). *P < 0.05, Student’s *t* test. (**b**) Methylation levels of CpG regions at the *TAF15* transcription start site. (**c**) *TAF15* protein levels in DTCs and untreated colonies derived from MKN45 cells by Western blot. The band intensities were relative to those of GAPDH. The numerical values are the relative intensity. (**d**) The expression level of *TAF15* mRNAs quantified using qRT-PCR in MKN45 cells treated with DMSO or α-AMA over a time course relative to respective *GAPDH* mRNA. ***P < 0.001, Student’s *t* test. (**e**) *TAF15* protein levels of MKN45 cells treated with DMSO or α-AMA over a time course by Western blot. The numerical band intensities were relative to those of respective GAPDH, and then adjusted to 1.0 in DMSO. (**f**) Both α-AMA treatment and *TAF15* siRNA transfection induces morphological changes in MKN45 cells. Scale bars = 20 μm. Corresponding box plots show length of cells (*n* = 20) in indicated conditions. **P < 0.01, ***P < 0.001, Student’s *t* test. (**g**) Colony formation assay of MKN45 cells transfected with two independent *TAF15*-targeting siRNAs (si-*TAF15*) or a scramble siRNA (Scr). MKN45 cells were transfected with siRNAs prior to colony formation in the presence (DTCs) and absence (untreated) of 0.2 μM CIS. si-*TAF15* #1 and #2 targeted the 3′-untranslated and coding regions, respectively. Each experiment was performed in triplicate. Error bars represent s.e.m.

We next screened the potential molecular target of α-AMA based on transcriptional DNA microarray data using MCF7 DTCs that exhibited a high CIS tolerance (Fig. 1b). Among the top 2.5% of specifically-induced genes in DTCs, we focused on *TAF15* because its gene product binds RNAPII^26^,^27^ (Fig. 6a). The promoter methylation status and protein expression level of *TAF15* in MKN45 DTCs were in good agreement with the gene expression data (Fig. 6b,c). *TAF15* encodes TATA-binding protein (TBP)-associated factor 15, which is a member of the TET (TLS/FUS, EWS, *TAF15*) family of RNA-binding proteins^27^. A recent study demonstrated that the *TAF15* protein binds the C-terminal domain of RNAPII more avidly than EWS and TLS/FUS proteins^28^. Our transcriptional screening with DTCs demonstrated that the level of *TAF15* was higher than EWS and TLS/FUS levels (Fig. 6a; Supplementary Fig. S11). The protein level of *TAF15* was higher in DTCs whereas EWS and TLS/FUS proteins did not show increased levels in DTCs (Fig. 6c; Supplementary Fig. S11). Thus, we postulate that *TAF15* is specifically involved in DTC formation among the TET family. The most abundant *TAF15*-associated RNA component has been reported to be U1 small-nuclear (sn)RNA, which is part of the splicing machinery^29^,^30^. The level of *TAF15*-associated U1 snRNA in DTCs and untreated colonies was not significantly different (Supplementary Fig. S12). These findings suggest that the U1 snRNA-binding capacity of *TAF15* is not predominantly involved in DTC formation.

We next focused on a causal link between *TAF15* upregulation and α-AMA sensitivity in DTC-forming cells. In response to α-AMA treatment, both *TAF15* mRNA and *TAF15* protein levels were decreased in MKN45 cells, suggesting that the RNAPII activity towards *TAF15* mRNA was inhibited by α-AMA (Fig. 6d,e). Notably, a similar morphological change was observed in MKN45 cells following both *TAF15* knockdown and α-AMA treatment (Fig. 6f). A subsequent colony formation assay revealed that *TAF15* knockdown suppressed the emergence of both DTCs and untreated colonies, suggesting that *TAF15* is a crucial target of α-AMA in the context of DTC suppression (Fig. 6g; Supplementary Fig. S13). In contrast, the overexpression of *TAF15* did not result in a greater number of DTCs compared to the mock control (Supplementary Figs S13 and S14). These results suggest that α-AMA treatment may present a therapeutic opportunity for diseases caused by DTCs, such as PC by chemotherapy, via inhibition of *TAF15* mRNA synthesis.

### α-AMA/CIS-combined treatment restraining *PC* in nude mice

**Figure 7:**
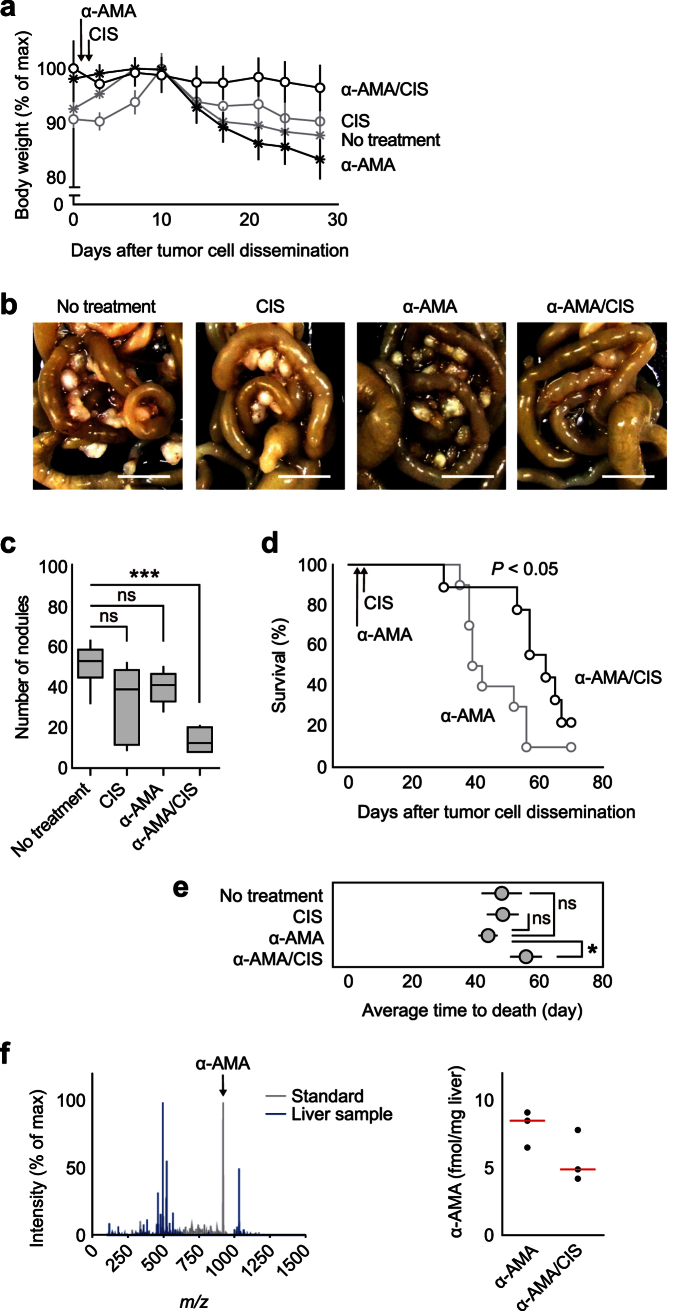
α-AMA/CIS-combined treatment restraining *PC* in nude mice. (**a**) Body weight changes of *PC* mice treated with α-AMA alone (α-AMA), CIS alone (CIS), serial administration of α-AMA and CIS (α-AMA/CIS) or no treatment. Each group of experiments was performed in a set of 16 biological replicates. Error bars represent s.e.m. (**b**) Representative images of disseminated and mature peritoneal nodules in mesentery tissue from no treatment, CIS, α-AMA, and α-AMA/CIS groups. Scale bars = 10 mm. (**c**) A comparison of the number of nodules in *PC* mice with no treatment and CIS, α-AMA, and α-AMA/CIS groups 28 days after MKN45 cell inoculation. Each group of experiments was performed in five or six biological replicates. ***P < 0.001, Student’s *t* test. ns, not significant. (**d**) Kaplan-Meier plots of *PC* mice treated with α-AMA and α-AMA/CIS. Each group of experiments was performed in a set of 10 biological replicates. Number of mice at risk *P* values were determined using the log-rank test. (**e**) A comparison of the average time to death of the no treatment, CIS, α-AMA, and α-AMA/CIS groups. The α-AMA/CIS group exhibited significantly longer time to death. Error bars represent ± SEM. *P < 0.05, Mann-Whitney *U* test. ns, not significant. (**f**) LC-MS analysis of α-AMA from the liver. MS peaks from α-AMA authentic standard and representative liver sample (left). MS peak at *m/z* 919.3619 was identified as α-AMA. α-AMA levels in liver tissues from the α-AMA and α-AMA/CIS groups (right). Red lines indicate the median values of individual liver samples for each condition. LC-MS, liquid chromatography-mass spectrometry; α-AMA, α-amanitin; CIS, cisplatin.

Selective suppression of DTC emergence by α-AMA *in vitro* offers a therapeutic opportunity to prevent PC. However, α-AMA ingestion was reported to be directly linked to death due to toxicities in the gastrointestinal tract, liver, and kidney^31^. Therefore, we evaluated both the therapeutic and toxicological properties of α-AMA using a PC model established by intraperitoneal inoculation of MKN45 cells followed by drug treatment. The body weights of mice after tumor cell inoculation decreased sharply starting on day 10 in α-AMA alone, CIS alone, and no treatment groups, whereas groups receiving serial administration of α-AMA and CIS maintained their body weight (Fig. 7a). The nodules spread mainly in the mesentery and ranged from 1 to 5 mm in diameter in mice from the α-AMA, CIS, and no treatment groups, while the majority of the mice in the α-AMA/CIS group exhibited very few nodules (Fig. 7b). In fact, the average number of nodules in the α-AMA/CIS group was significantly decreased, although CIS and α-AMA single administration groups exhibited a similar suppression effect, but to a lesser degree (Fig. 7c). The overall survival of mice in the α-AMA/CIS group was significantly longer than for those in the α-AMA group, suggesting that the α-AMA toxicity was reduced by CIS combination (Fig. 7d). A comparison of the average time to death of the four treatment groups revealed that only the α-AMA/CIS group exhibited a significant prolongation in overall survival (Fig. 7e). The extension of the average time to death with α-AMA/CIS treatment was approximately 10 days within the 70-day period compared to other treatment groups. Subsequently, we performed pathological examination and a liquid chromatography-mass spectrometry (LC-MS) analysis to assess the effect of CIS administration on α-AMA toxicity. Pathological examination of the liver and kidney at 48 hours after α-AMA administration revealed no remarkable findings at a dose of 0.4 mg/kg, which was used in the PC mouse model (Supplementary Fig. S15). Substantial pathological changes were observed only at higher doses (1.2 and 3.6 mg/kg). An LC-MS analysis for α-AMA concentration revealed that a higher dose than 3.6 mg/kg was needed to detect α-AMA from the liver samples (Supplementary Fig. S16). Interestingly, the α-AMA level in the liver was 42% lower in the α-AMA/CIS group compared to the α-AMA single administration group, suggesting that CIS may inhibit α-AMA accumulation in the liver (Fig. 7f). In principle, these results indicate that: (i) the number of nodules in mesentery may be a surrogate marker of overall survival in PC; and (ii) α-AMA possesses the capacity to prevent cancer relapse due to PC when it is administered with CIS. Taken together, the combination of α-AMA and CIS appears to elicit the potential suppression effect of α-AMA for PC while also reducing its toxicity.

## Discussion

We used DTCs as an *in vitro* model for relapsed cancer cells, which are subpopulations that survive in the presence of anticancer drugs. Each DTC can be initiated from either a single cell or an extremely small number of cells, which mimics the process of cancer relapse in humans after curative surgery followed by adjuvant chemotherapy. Proteomic characterization of DTCs using CoLA technology showed that there were two distinct groups of cells based on stemness- and epithelium-associated protein expression, while the expression of a set of genes known to be associated with cellular pluripotency appeared to affect colony formation^19^,^32^. These results led us to hypothesize that DTC formation may be suppressed by inhibiting fundamental transcriptional machinery at a relatively early stage of DTC formation and prior to protein synthesis. Consistent with the finding that transcription is involved in DTC formation, the RNAPII inhibitor α-AMA demonstrated a significant reduction in DTC formation. The mechanism of action for α-AMA appeared to involve down-regulation of *TAF15*, which participates in RNAPII-dependent transcriptional machinery^27^. Importantly, α-AMA/CIS exhibited an inhibitory effect on *PC in vivo*, suggesting that α-AMA may be used for the prevention of *PC* with conventional chemotherapy.

A variety of mechanisms for drug tolerance have been proposed, including target gene mutation^33^, adaptive resistance^34^,^35^, selection of intrinsically resistant populations^36^, and epigenetic alterations^6^. Empirical clinical observations suggest that cancer relapse does not always result from a specific population that acquired drug-tolerant properties in response to administered anticancer drugs, but may instead be due to undefined populations among total heterogeneous cancer cell populations that happen to have drug tolerance^37^ or acute adaptive responses to a particular drug^35^. In fact, complete suppression of cancer relapse is often extremely challenging because of difficulties in: (i) selectively targeting cancer initiating cells; (ii) defining effective drug combinations; and (iii) selecting inhibitors that affect the fundamental components of cell division. Although there are no definitive clinical guidelines for all relapsed situations, conventional DNA damaging agents or taxanes are often used for *PC* patients^38^,^39^. In the present study, we used CIS in a combinational approach with α-AMA to elucidate its therapeutic potential based on molecular fraction screening for colony formation inhibition. α-AMA, which is a bicyclic octapeptide produced by the basidiomycetes mushroom, *Amanita phalloides*, is a potent inhibitor of RNAPII^23^. Very few therapeutic opportunities that exploit α-AMA have been pursued because of its high gastrointestinal tract, liver, and renal toxicities^31^. However, recent development of a cancer immunotherapy using an anti-EPCAM monoclonal antibody conjugated with α-AMA demonstrated clear growth suppression effects in a human pancreatic cancer mouse skin xenograft model without systemic side effects even at a dose of 0.1 mg/kg^40^. Our model mimics human *PC* after apparently “curative removal” of solid tumors in the abdomen. The combinational therapy of α-AMA and CIS indicates that inhibition of RNAPII is critical for cells to develop as a mass in the peritoneum. Importantly, our treatment with α-AMA did not show significant lethal side effects despite peritoneal injections at a dose of 0.4 mg/kg. The mouse median lethal dose for α-AMA was estimated to be higher than 1.2 μg/kg in our validation. Importantly, α-AMA accumulation in the mouse liver was inhibited by CIS administration. These results suggest that the α-AMA dose used in the present study is tolerable and sufficient for an antitumor effect.

While searching for molecular targets of α-AMA, the *TAF15* gene, which encodes a transcription factor that binds RNAPII directly, was identified. *TAF15* was first isolated as a TATA-binding protein-associated factor (TAF) in the general transcription factor IID complex that initiates RNAPII-dependent transcriptional machinery and pre-mRNA splicing^27^,^41^. A protein family consisting of translocated in liposarcoma or fusion in sarcoma (TLS/FUS), Ewing’s sarcoma (EWS), and *TAF15* is called the TET (TLS/FUS, EWS, *TAF15*) family and has various roles in gene expression^42^. *TAF15* is involved in chromosomal translocations in acute leukemia^43^ and extraskeletal myxoid sarcomas^44^, resulting in fusion proteins that may have oncologic potential through enhanced cell spreading and adhesion^45^. For *TAF15*-targeted immunotherapy, a human IgG PAT-BA4 was developed against the tumor-specific *TAF15* variant^46^. Although the therapeutic effect of PAT-BA4 has been limited *in vitro*, *TAF15* targeting therapy has demonstrated anti-adhesion and anti-migration effects in cancer cells, which indicates the importance of *TAF15* for increased malignant potential. In the present study we demonstrated that *TAF15* mRNA was one of the crucial components for initiating DTC formation. However, from the transcriptional profiling of DTCs, the formation and establishment of DTCs does not appear to be a process that requires merely a small number of specific genes or proteins. In fact, at least several hundred genes lie downstream of *TAF15*^47^. Hence, we suggest that an agent that can inhibit mRNA synthesis over a wide spectrum, such as α-AMA, may be more appropriate for preventing cancer relapse than those compounds that target a single molecule. From a therapeutic point-of-view, whether α-AMA doses having sufficient antitumor effects would be tolerable in humans either by ingestion or peritoneal injection remains to be determined^48^.

In summary, our study demonstrated that RNAPII-dependent mRNA synthesis is a fundamental process for DTC formation and establishment of *PC*. These findings provide the foundation for a promising strategy to prevent cancer relapse after apparently successful surgical and chemotherapeutic treatments.

## Materials and Methods

### Cell Culture

HCT116, HT29, HeLa, MCF7 and MKN45 cells were obtained from the RIKEN Cell Bank. All five cell lines were grown in RPMI 1640 media (Life Technologies, Carlsbad, CA, USA) supplemented with 10% fetal bovine serum (FBS) (Life Technologies), and cultured at 37 °C in a humidified incubator supplied with 5% CO_2_.

### Drugs

CIS and DTX were purchased through Nippon Kayaku (Tokyo, Japan) and Sanofi KK (Tokyo, Japan), respectively; TSA was purchased through Wako (Osaka, Japan). AMD, α-AMA and CHX were purchased through Sigma-Aldrich (St. Louis, MO, USA); GEF and SOR were purchased through Tocris (Bristol, UK) and Cell Signaling Technology (Danvers, MA, USA), respectively.

### Growth Suppression and Colony formation Assay

For growth suppression assay, cells were plated in a 96-well plate at 3.2 − 12.8 × 10^4 ^cells/cm^2^. The cell viability was determined using CCK-8 (Dojindo, Kumamoto, Japan) and a TriStar LB 941 microplate reader (Berthold Technologies, Bad Wildbad, Germany). For colony formation assay, drugs or compounds were dispensed into 48-well plates before cell dissemination. Cells were then plated at 1.3 − 2.6 × 10^2 ^cells/cm^2^. Colonies were stained with 0.1% (w/v) crystal violet.

### CoLA

For colony lysate preparation, a total of 2400 colonies were isolated individually using suction produced by a micropipette, and individual cell pellets were lysed with 10 μl Pink Buffer as previously described^14^. The lysates were dotted onto nitrocellulose membranes attached to a glass slide using a 2470 Arrayer (Aushon Biosystems, Billerica, MA, USA). CoLAs were incubated with specific primary antibodies (Supplementary Table S2) followed by analysis with a Catalyzed Signal Amplification System (Dako Japan, Tokyo, Japan). For detection of total protein levels, CoLAs were stained with colloidal gold (Bio-Rad, Hercules, CA, USA). An optical flatbed GT-X970 scanner (EPSON, Suwa, Japan) with the resolution set to 16-bit, 2400 dpi was used for image acquisition. To prevent skewing of image intensities, a Wedge Density Strip (Danes-Picta, Praha, Czech Republic) was used for readout range calibration^49^. Spot images were converted to raw pixel values using P-SCAN software^50^ or WinPscan ( http://abs.cit.nih.gov/pscan; http://www.nishizukalab.org).

### Xenografts

For tumorigenicity tests, six colonies (untreated) and DTCs derived from MKN45 cells were individually injected subcutaneously into the left and right side of the backs of six 6-week-old female nude mice (BALB/cAjcl-nu/nu, CLEA Japan, Tokyo, Japan). These mice were monitored for 49 days after the inoculation or until tumors reached 10 mm in the largest diameter, and were then euthanized. For the PC model, 1.0 × 106 MKN45 cells were injected intraperitoneally into six 6-week-old female nude mice (BALB/cAjcl-nu/nu, CLEA Japan). Mice were then treated with CIS (4.0 mg/kg, intraperitoneal administration) or a combination of CIS and α-AMA (0.4 mg/kg, intraperitoneal administration). For the combination treatment, α-AMA was given 24 hours before CIS. Body weight was monitored for 28 days after the treatment. All experiments were performed in accordance with the approval of the Iwate Medical University Ethical Committee for Animal Experiment Regulation (23-067, 25-023, and 26-004).

### Visualized Cell Cycle Analysis

The cell cycle in sheets and colonies of HeLa cells was monitored using the Fucci system (HeLa.S-Fucci2)^11^. In this system, cells in the S/G_2_/M or G_1_ phase express Venus-hGem or mCherry-Cdt1 protein, respectively. HeLa.S-Fucci2 cells were seeded as cell sheets (3.0 × 10^4 ^cells/cm^2^) or as colonies (1.0 × 10^2 ^cells/cm^2^). Cells were treated for 48 hours with CIS GI_50_ for sheets, and for 13 days with CIS CoI_50_ for colonies. Conditions for untreated cells were used as a control. For quantification of the fluorescence intensity of individual cells, cell sheets and colonies were diluted to single cell suspensions, and the fluorescence intensity was quantified using a Tali Image-Based Cytometer (Life Technologies, Carlsbad, CA, USA). The 75^th^ percentile of signal in untreated or CIS-treated sheets was defined as Venus- or mCherry-positive cells, respectively (Supplementary Fig. S3).

### DNA Microarray

Total RNA was extracted from two biological replicates of bulk untreated colonies and DTCs of MCF7 emerged in the presence of 0.8 μM CIS using the RNeasy Kit (QIAGEN, Tokyo, Japan). Gene expression profiling was performed according to the manufacturer’s instructions (SurePrint G3 Human Gene Expression 8 × 60 K microarrays, Agilent Japan, Tokyo, Japan). Raw data were normalized by dividing each probe signal by the 75^th^ percentile of the entire signal. For identification of the *TAF15* gene, gene ontology analysis was performed using Database for Annotation, Visualization and Integrated Discovery (DAVID) software tools ( http://david.abcc.ncifcrf.gov/). The DNA microarray data (GSE65419) is available via Gene Expression Omnibus (http://www.ncbi.nlm.nih.gov/geo/).

### Methylated DNA Immunoprecipitation Combined with Microarray

Methylated DNA immunoprecipitation combined with microarray (MeDIP-chip) analysis was performed as described previously^51^,^52^. Genomic DNA was extracted from bulk untreated colonies and DTCs of MKN45 emerged in the presence of 0.2 μM CIS using the DNeasy Kit (QIAGEN). Fluorescent labeling, microarray hybridization, washing and scanning were performed according to the manufacturer’s instructions (Human Meth 385K Prom Plus CpG Arrays, NimbleGen, Madison, WI, USA). Immunoprecipitated DNA samples from untreated colonies and DTCs were labeled with Cy3 and Cy5, respectively. Each raw data point was first normalized by *Z* score transformation, and then calculated as a Cy5/Cy3 log_2_ ratio. Promoter regions with differentially methylated CpG sites were identified using SignalMap Software version 2.0 (NimbleGen). The MeDIP-chip data (GSE69203) is available via Gene Expression Omnibus (http://www.ncbi.nlm.nih.gov/geo/).

### Immunocytochemistry

Cells were seeded onto 96- or 48-well plates and incubated for 13 days in the presence or absence of 0.2 μM CIS. Colonies were then fixed in 4% paraformaldehyde (PFA) for 15 min at room temperature and stained with 4′,6-diamidino-2-phenylindole (DAPI) for 20 min. After washing, cells were blocked in 5% BSA and 0.1% Tween 20 before staining with anti-CD44 antibody (156-3C11, Cell Signaling Technology, Danvers, MA, USA) at 4 °C overnight. After washing with phosphate buffered saline (PBS), CF488A-conjugated secondary antibodies (Biotium, Hayward, CA, USA) were applied for 1 hour at room temperature. After washing, wells were filled with PBS. Images were collected on an IN Cell Analyzer 2000 automated microscope and deconvoluted using IN Cell Analyzer 2000 software (GE Healthcare). The deconvoluted image projections were analyzed using Developer (GE Healthcare) to identify colonies.

### ROS Detection

For ROS detection, colonies were incubated with the cell-permeable fluorescent indicator CellROX Deep Red (Life Technologies) and Hoechst 33342 at 37 °C for 30 min. After washing, wells were filled with PBS and images were collected as described in the Immunocytochemistry section. Immunocytochemistry for CD44 was performed after ROS detection.

### qRT-PCR

For single-colony analysis in Fig. 4e, total RNA was extracted from 10 individually isolated untreated colonies and DTCs derived from MKN45 cells using the RNeasy kit (QIAGEN). A 1-mm diameter single MKN45 colony (approximately 1.0 × 10^4^ cells) yielded approximately 100 ng of total RNA. cDNA was synthesized from 50 ng total RNA in a 10 μl reaction volume using PrimeScript RT Master Mix (TaKaRa Bio, Otsu, Japan).

For the time course analysis in Fig. 6d, total RNA was extracted from 1.0 × 10^6^ MKN45 cells treated with DMSO or α-AMA for 0, 4, 24, and 48 hours. cDNA was synthesized from 500 ng total RNA in a 10 μl reaction volume using PrimeScript RT Master Mix (TaKaRa Bio). Quantitative RT-PCR (qRT-PCR) was performed using the LightCycler Nano System (Roche, Mannheim, Germany). Primer sequences are listed in Supplementary Table S3.

### Western Blotting

Cells were harvested by trypsinization, and lysed with Pink Buffer as previously described^14^. Ten micrograms of lysate was resolved on NuPAGE 4-12% Bis-Tris gels (Life Technologies) and electrophoretically transferred to a nitrocellulose membrane using iBlot Dry Blotting System (Life Technologies). Membranes were blocked for 1 hr in 5% BSA in Tris-buffered saline with Tween 20 (TBST, Dako), and then incubated overnight with specific primary antibodies in 5% BSA in TBST. Horseradish peroxidase (HRP)-conjugated anti-rabbit IgG secondary antibody (1:2000, Cell Signaling Technologies) was diluted in 5% nonfat dry milk in TBST. Chemiluminescence detection reagents were incubated with the membrane, and an image was acquired using an Image Quant LAS500 (GE Healthcare). Band intensities were quantified using ImageJ version 1.49q ( http://rsbweb.nih.gov/ij/). Primary antibodies used in this analysis are shown in Supplementary Table S2.

### Knockdown of *TAF15*

For *TAF15* knockdown, 1.0 × 10^5^ MKN45 cells were transfected with 5 nM siRNA using Lipofectamine RNAiMAX (Life technologies) for 48 hours at 37 °C in a humidified incubator supplied with 5% CO_2_. Morphological changes were observed after 48 hours. To assess the effect of *TAF15* knockdown on DTC generation, cells were disseminated in the presence or absence of 0.2 μM CIS at low density (2.0 × 10^2 ^cells/cm^2^). Approximately 100 colonies per well from the siRNA-transfected cells appeared in the control (i.e., without CIS) 13 days after the dissemination. Colony counting was performed as described in the Colony Formation Assay section. siRNAs sequences were described elsewhere^47^.

## Additional Information

**How to cite this article**: Kume, K. *et al*. α-Amanitin Restrains Cancer Relapse from Drug-Tolerant Cell Subpopulations via *TAF15*. *Sci. Rep.* **6**, 25895; doi: 10.1038/srep25895 (2016).

## Supplementary Material

Supplementary Information

## References

[b1] Sakuramoto S. . Adjuvant chemotherapy for gastric cancer with S-1, an oral fluoropyrimidine. N Engl J Med 357, 1810–1820 (2007). 17978289 10.1056/NEJMoa072252

[b2] Paoletti X. . Benefit of adjuvant chemotherapy for resectable gastric cancer: a meta-analysis. JAMA 303, 1729–1737 (2010). 20442389 10.1001/jama.2010.534

[b3] Yachida S. . Distant metastasis occurs late during the genetic evolution of pancreatic cancer. Nature 467, 1114–1117 (2010). 20981102 10.1038/nature09515 PMC3148940

[b4] Campbell P. J. . The patterns and dynamics of genomic instability in metastatic pancreatic cancer. Nature 467, 1109–1113 (2010). 20981101 10.1038/nature09460 PMC3137369

[b5] O’Connell M. J. . Improving adjuvant therapy for rectal cancer by combining protracted-infusion fluorouracil with radiation therapy after curative surgery. N Engl J Med 331, 502–507 (1994). 8041415 10.1056/NEJM199408253310803

[b6] Sharma S. V. . A chromatin-mediated reversible drug-tolerant state in cancer cell subpopulations. Cell 141, 69–80 (2010). 20371346 10.1016/j.cell.2010.02.027 PMC2851638

[b7] Ramaswamy S., Ross K. N., Lander E. S. & Golub T. R. A molecular signature of metastasis in primary solid tumors. Nat Genet 33, 49–54 (2003). 12469122 10.1038/ng1060

[b8] Kreso A. . Variable clonal repopulation dynamics influence chemotherapy response in colorectal cancer. Science 339, 543–548 (2013). 23239622 10.1126/science.1227670 PMC9747244

[b9] Baylin S. B. Resistance, epigenetics and the cancer ecosystem. Nat Med 17, 288–289 (2011). 21383739 10.1038/nm0311-288

[b10] Singh S. K. . Identification of a cancer stem cell in human brain tumors. Cancer Res 63, 5821–5828 (2003). 14522905

[b11] Sakaue-Sawano A., Kobayashi T., Ohtawa K. & Miyawaki A. Drug-induced cell cycle modulation leading to cell-cycle arrest, nuclear mis-segregation, or endoreplication. BMC cell biology 12, 2 (2011). 21226962 10.1186/1471-2121-12-2 PMC3277280

[b12] Zhao B., Hemann M. T. & Lauffenburger D. A. Intratumor heterogeneity alters most effective drugs in designed combinations. Proc Natl Acad Sci USA 111, 10773–10778 (2014). 25002493 10.1073/pnas.1323934111 PMC4115561

[b13] Spurrier B., Washburn F. L., Asin S., Ramalingam S. & Nishizuka S. Antibody screening database for protein kinetic modeling. Proteomics 7, 3259–3263 (2007). 17708592 10.1002/pmic.200700117

[b14] Nishizuka S. . Proteomic profiling of the NCI-60 cancer cell lines using new high-density reverse-phase lysate microarrays. Proc Natl Acad Sci USA 100, 14229–14234 (2003). 14623978 10.1073/pnas.2331323100 PMC283574

[b15] Gallmeier E. . Inhibition of ataxia telangiectasia- and Rad3-related function abrogates the *in vitro* and *in vivo* tumorigenicity of human colon cancer cells through depletion of the CD133(+) tumor-initiating cell fraction. Stem Cells 29, 418–429 (2011). 21308861 10.1002/stem.595

[b16] Vermeulen L. . Single-cell cloning of colon cancer stem cells reveals a multi-lineage differentiation capacity. Proc Natl Acad Sci USA 105, 13427–13432 (2008). 18765800 10.1073/pnas.0805706105 PMC2533206

[b17] Ishimoto T. . CD44 variant regulates redox status in cancer cells by stabilizing the xCT subunit of system xc(-) and thereby promotes tumor growth. Cancer Cell 19, 387–400 (2011). 21397861 10.1016/j.ccr.2011.01.038

[b18] Adewumi O. . Characterization of human embryonic stem cell lines by the International Stem Cell Initiative. Nat Biotechnol 25, 803–816 (2007). 17572666 10.1038/nbt1318

[b19] Okita K., Ichisaka T. & Yamanaka S. Generation of germline-competent induced pluripotent stem cells. Nature 448, 313–317 (2007). 17554338 10.1038/nature05934

[b20] Ohnishi K. . Premature termination of reprogramming *in vivo* leads to cancer development through altered epigenetic regulation. Cell 156, 663–677 (2014). 24529372 10.1016/j.cell.2014.01.005

[b21] Bolden J. E., Peart M. J. & Johnstone R. W. Anticancer activities of histone deacetylase inhibitors. Nat Rev Drug Discov 5, 769–784 (2006). 16955068 10.1038/nrd2133

[b22] Bensaude O. Inhibiting eukaryotic transcription: Which compound to choose? How to evaluate its activity? Transcription 2, 103–108 (2011). 21922053 10.4161/trns.2.3.16172 PMC3173647

[b23] Lindell T. J., Weinberg F., Morris P. W., Roeder R. G. & Rutter W. J. Specific inhibition of nuclear RNA polymerase II by alpha-amanitin. Science 170, 447–449 (1970). 4918258 10.1126/science.170.3956.447

[b24] Schneider-Poetsch T. . Inhibition of eukaryotic translation elongation by cycloheximide and lactimidomycin. Nat Chem Biol 6, 209–217 (2010). 20118940 10.1038/nchembio.304 PMC2831214

[b25] Shi L. . Histone demethylase JMJD2B coordinates H3K4/H3K9 methylation and promotes hormonally responsive breast carcinogenesis. Proc Natl Acad Sci USA 108, 7541–7546 (2011). 21502505 10.1073/pnas.1017374108 PMC3088624

[b26] Bertolotti A. . EWS, but not EWS-FLI-1, is associated with both TFIID and RNA polymerase II: interactions between two members of the TET family, EWS and hTAFII68, and subunits of TFIID and RNA polymerase II complexes. Mol Cell Biol 18, 1489–1497 (1998). 9488465 10.1128/mcb.18.3.1489 PMC108863

[b27] Bertolotti A., Lutz Y., Heard D. J., Chambon P. & Tora L. hTAF(II)68, a novel RNA/ssDNA-binding protein with homology to the pro-oncoproteins TLS/FUS and EWS is associated with both TFIID and RNA polymerase II. EMBO J 15, 5022–5031 (1996). 8890175 PMC452240

[b28] Kwon I. . Phosphorylation-regulated binding of RNA polymerase II to fibrous polymers of low-complexity domains. Cell 155, 1049–1060 (2013). 24267890 10.1016/j.cell.2013.10.033 PMC4010232

[b29] Jobert L. . Human U1 snRNA forms a new chromatin-associated snRNP with *TAF15*. EMBO Rep 10, 494–500 (2009). 19282884 10.1038/embor.2009.24 PMC2680868

[b30] Kugel J. F. & Goodrich J. A. In new company: U1 snRNA associates with *TAF15*. EMBO Rep 10, 454–456 (2009). 19373252 10.1038/embor.2009.65 PMC2680880

[b31] Ward J., Kapadia K., Brush E. & Salhanick S. D. Amatoxin poisoning: case reports and review of current therapies. J Emerg Med 44, 116–121 (2013). 22555054 10.1016/j.jemermed.2012.02.020

[b32] Yu J. . Induced pluripotent stem cell lines derived from human somatic cells. Science 318, 1917–1920 (2007). 18029452 10.1126/science.1151526

[b33] Holohan C., Van Schaeybroeck S., Longley D. B. & Johnston P. G. Cancer drug resistance: an evolving paradigm. Nat Rev Cancer 13, 714–726 (2013). 24060863 10.1038/nrc3599

[b34] Redmond K. M., Wilson T. R., Johnston P. G. & Longley D. B. Resistance mechanisms to cancer chemotherapy. Front Biosci 13, 5138–5154 (2008). 18508576 10.2741/3070

[b35] Muranen T. . Inhibition of PI3K/mTOR leads to adaptive resistance in matrix-attached cancer cells. Cancer Cell 21, 227–239 (2012). 22340595 10.1016/j.ccr.2011.12.024 PMC3297962

[b36] Valent P. . Cancer stem cell definitions and terminology: the devil is in the details. Nat Rev Cancer 12, 767–775 (2012). 23051844 10.1038/nrc3368

[b37] Gupta P. B. . Stochastic state transitions give rise to phenotypic equilibrium in populations of cancer cells. Cell 146, 633–644 (2011). 21854987 10.1016/j.cell.2011.07.026

[b38] Sloothaak D. . Intraperitoneal chemotherapy as adjuvant treatment to prevent peritoneal carcinomatosis of colorectal cancer origin: a systematic review. British journal of cancer 111, 1112–1121 (2014). 25025964 10.1038/bjc.2014.369 PMC4453838

[b39] Ansaloni L. . Pharmacokinetics of concomitant cisplatin and paclitaxel administered by hyperthermic intraperitoneal chemotherapy to patients with peritoneal carcinomatosis from epithelial ovarian cancer. British journal of cancer , 112, 306–312 (2015). 25461804 10.1038/bjc.2014.602 PMC4453456

[b40] Moldenhauer G. . Therapeutic potential of amanitin-conjugated anti-epithelial cell adhesion molecule monoclonal antibody against pancreatic carcinoma. J Natl Cancer Inst 104, 622–634 (2012). 22457476 10.1093/jnci/djs140

[b41] Calvio C., Neubauer G., Mann M. & Lamond A. I. Identification of hnRNP P2 as TLS/FUS using electrospray mass spectrometry. RNA 1, 724–733 (1995). 7585257 PMC1369314

[b42] Tan A. Y. & Manley J. L. The TET family of proteins: functions and roles in disease. J Mol Cell Biol 1, 82–92 (2009). 19783543 10.1093/jmcb/mjp025 PMC2905059

[b43] Martini A. . Recurrent rearrangement of the Ewing’s sarcoma gene, EWSR1, or its homologue, *TAF15*, with the transcription factor CIZ/NMP4 in acute leukemia. Cancer Res 62, 5408–5412 (2002). 12359745

[b44] Sjogren H., Meis-Kindblom J., Kindblom L. G., Aman P. & Stenman G. Fusion of the EWS-related gene TAF2N to TEC in extraskeletal myxoid chondrosarcoma. Cancer Res 59, 5064–5067 (1999). 10537274

[b45] Andersson M. K. . The multifunctional FUS, EWS and *TAF15* proto-oncoproteins show cell type-specific expression patterns and involvement in cell spreading and stress response. BMC Cell Biol 9, 37 (2008). 18620564 10.1186/1471-2121-9-37 PMC2478660

[b46] Schatz N., Brandlein S., Ruckl K., Hensel F. & Vollmers H. P. Diagnostic and therapeutic potential of a human antibody cloned from a cancer patient that binds to a tumor-specific variant of transcription factor *TAF15*. Cancer Res 70, 398–408 (2010). 20048082 10.1158/0008-5472.CAN-09-2186

[b47] Ballarino M. . *TAF15* is important for cellular proliferation and regulates the expression of a subset of cell cycle genes through miRNAs. Oncogene 32, 4646–4655 (2013). 23128393 10.1038/onc.2012.490

[b48] Mengs U., Pohl R. T. & Mitchell T. Legalon(R) SIL: the antidote of choice in patients with acute hepatotoxicity from amatoxin poisoning. Curr Pharm Biotechnol 13, 1964–1970 (2012). 22352731 10.2174/138920112802273353 PMC3414726

[b49] Nishizuka S., Washburn N. R. & Munson P. J. Evaluation method of ordinary flatbed scanners for quantitative density analysis. Biotechniques 40, 442, 444, 446 passim (2006). 10.2144/000112144 16629390

[b50] Carlisle A. J. . Development of a prostate cDNA microarray and statistical gene expression analysis package. Mol Carcinog 28, 12–22 (2000). 10820484

[b51] Wilson I. M. . Epigenomics: mapping the methylome. Cell Cycle 5, 155–158 (2006). 16397413 10.4161/cc.5.2.2367

[b52] Zhang X. . Genome-wide high-resolution mapping and functional analysis of DNA methylation in arabidopsis. Cell 126, 1189–1201 (2006). 16949657 10.1016/j.cell.2006.08.003

