# Supplementary material for: α-Amanitin Restrains Cancer Relapse from Drug-Tolerant Cell Subpopulations via TAF15
Source: Sci Rep. 2016 May 16;6:25895. doi: 10.1038/srep25895 (PMC4867652; doi:10.1038/srep25895)
Supplement: Supplementary Information [file srep25895-s1.pdf]

Supplementary Information

α-Amanitin Restrains Cancer Relapse from Drug-Tolerant Cell Subpopulations via *TAF15*

Kohei Kume[1,2,3], Miyuki Ikeda[1], Sawako Miura[1], Kohei Ito[1], Kei A. Sato[1], Yukimi Ohmori[1,2], Fumitaka Endo[1], Hirokatsu Katagiri[1], Kaoru Ishida[1], Chie Ito[1], Takeshi Iwaya[1], and Satoshi S. Nishizuka[1,2,3*].

[1]Molecular Therapeutics Laboratory, Department of Surgery, Iwate Medical University School of Medicine, Morioka, Iwate 020-8505, Japan.

[2]MIAST (Medical Innovation by Advanced Science and Technology) project, Iwate Medical University School of Medicine, Morioka, Iwate 020-8505, Japan.

[3]Institute of Biomedical Science, Iwate Medical University, Yahaba, Iwate 028-3694, Japan.

*Correspondence: Satoshi S. Nishizuka, Molecular Therapeutics Laboratory, Department of Surgery, Iwate Medical University School of Medicine, 19-1 Uchimaru, Morioka, Iwate, Japan, 020-8505. Phone: 81-19-651-5111; Fax: 81-19-651-7166; E-mail: snishizu@iwate-med.ac.jp.

Supplementary Materials and Methods

Growth Suppression Assay

Cells were plated in a 96-well plate at high density ($3.2\text{-}12.8 \times 10^4$ cells/cm^2) and allowed to adhere for 24 hours. Cells were then treated with eight 10-fold serial dilutions of drugs (starting concentrations are shown in Supplementary Fig. S1b). Intracellular dehydrogenase activity in viable cells was determined using CCK-8 (Dojindo, Kumamoto, Japan) and a TriStar LB 941 microplate reader (Berthold Technologies, Bad Wildbad, Germany). Fifty percent growth inhibition concentrations (GI_{50}) were calculated using GraphPad Prism software.

Colony Formation Assay

Before cell dissemination, eight 10-fold serial dilutions of drugs or compounds (starting concentrations are shown in Supplementary Fig. S1b), including no drug controls, were dispensed into 48-well plates. Cells were then plated in the prepared 48-well plates at low density ($1.3\text{-}2.6 \times 10^2$ cells/cm^2). Approximately 50 colonies per well appeared in the no drug control within 8-21 days after dissemination. Colonies were then stained with 0.1% (w/v) crystal violet. The number of colonies was counted manually or automatically using an IN Cell 2000 device (GE Healthcare Japan, Tokyo, Japan). Fifty percent colony formation inhibition concentrations (CoI_{50}) were calculated using GraphPad Prism software.

Generation of DTCs for CoLA

Before cell dissemination, six 10-fold serial dilutions of drugs (starting concentrations were shown in Supplementary Table S1), including no drug controls, were dispensed into 6-well plates. Cells were then plated in the prepared 6-well plates at low density (10-20 cells/cm^2). Approximately fifty colonies per well appeared in the no drug control wells within 8-21 days after dissemination.

Colony formation assay-based compound screening

Compound libraries that provided by Screening Committee of Anticancer Drugs (SCAD) were used in this study. Colony formation assay was performed in a 96-well format. Cells were seeded at a density of 50 cells per well in the presence of compound (1.0 µM). Approximately 20 colonies per well appeared in the no drug control within 13 days after cell dissemination. Colonies were stained with 0.1% (w/v) crystal violet. Crystal violet-stained colonies were detected automatically using an IN Cell 2000 device (GE Healthcare).

Overexpression of TAF15

For TAF15 overexpression, a TAF15 expression plasmid (HaloTag ORF clone pFN21AE2015, Kazusa DNA research institute, http://www.biosupport.kazusa.or.jp/english/sub_center1/) was used. MKN45 cells were plated at a density of 1.0×10^5 per 15 mm well, and transfected with 1 µg pFN21AE2015

using Lipofectamine 3000 (Life technologies) for 48 hours at 37°C in a humidified incubator supplied with 5% CO_2. DTC generation and colony counting was performed as described in the Materials and Methods section.

Co-Immunoprecipitation of U1-TAF15 complex

An anti-TAF15 antibody or control mouse IgG antibody were diluted in Tris-buffered saline (TBS) (50 mM Tris-HCl/150 mM NaCl, pH 8.0) and incubated 15 min at room temperature with 5 µl (25 µl medium slurry) of Protein G Mag Sepharose (GE Healthcare). DTCs were formed in 75 cm^2 flask in the presence of 0.2 µM CIS by seeding MKN45 cells at low density (100 cells/cm^2). Nuclear extracts (NEs) were prepared using the Nuclear Complex Co-IP Kit (Active Motif, Tokyo, Japan) and diluted in TBS. A U1-TAF15 complex was isolated from the NEs by incubating with antibody/sepharose suspension overnight at 4°C. Sepharose beads were washed three times with TBS. The beads and input NEs were treated with Proteinase K (QIAGEN, Tokyo, Japan) and then DNase I (TaKaRa Bio, Otsu, Japan). RNAs were extracted with phenol/chloroform/isoamylalchole (25:24:1) and recovered by ethanol precipitation with ethachinmate (Nippon Gene, Tokyo, Japan) after each enzymatic reaction. One hundred ng input RNA and the same volume of immunoprecipitated samples were used for quantitative RT-PCR (qRT-PCR). qRT-PCR was performed as described in the Materials and Methods section.

LC-MS analysis

Ten-week-old female nude mice (BALB/cAjcl-nu/nu, CLEA Japan) were treated with α-AMA (0.4, 1.2, 3.6 mg/kg, intraperitoneal administration) or a combination of α-AMA and CIS (4.0 mg/kg, intraperitoneal administration). CIS was given 24 hours after α-AMA administration. These mice were euthanized at 48 hours after the α-AMA administration, and 50-150 mg of liver samples were collected. These liver samples were completely powdered under liquid nitrogen and stored at -80°C for further use. The liver powder was homogenized in methanol, and the supernatant was used for mass spectrometry analysis using LC-Orbitrap-MS (Thermo Scientific, Waltham, MA). The obtained data were analyzed using SIEVE software version 2.2.57 (Thermo Scientific). Authentic standard of α-AMA was purchased through Sigma-Aldrich (St. Louis, MO, USA), and the MS peak was identified by reference to the Kyoto Encyclopedia of Genes and Genomes (KEGG) database. All animal procedures were performed in accordance with the approval of the Iwate Medical University Ethical Committee for Animal Experiment Regulation (23-067, 25-023, and 26-004).

Supplementary Figures

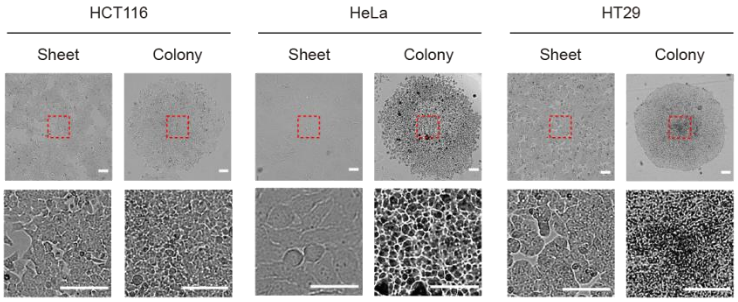

Figure S1. Morphology of a sheet and representative colony of HCT116, HeLa and HT29 cells. Scale bars = 100 µm. The area enclosed by the square in the top panels is shown at higher magnification in the bottom panel.

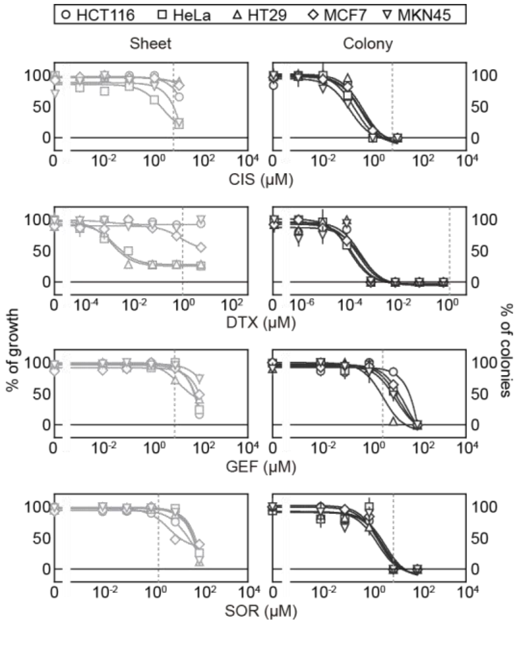

Figure S2. Drug dose-response curves of each cell line for a sheet and colony. Dotted lines indicate the peak plasma concentrations of the respective drugs in humans. All experiments were performed in triplicate. Error bars represent s.e.m.

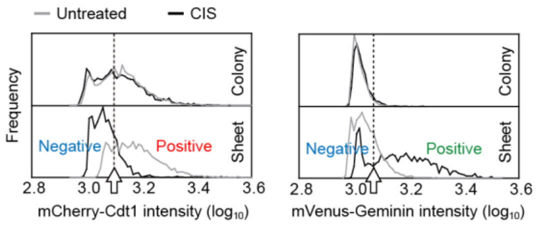

Figure S3. Determination of Cdt1- and Geminin-positive cells of sheet- and colony-derived cells using HeLa.S-Fucci2. Arrows indicate the 75th percentile of mCherry (red) and mVenus (green) signals in HeLa.S-Fucci2 sheet.

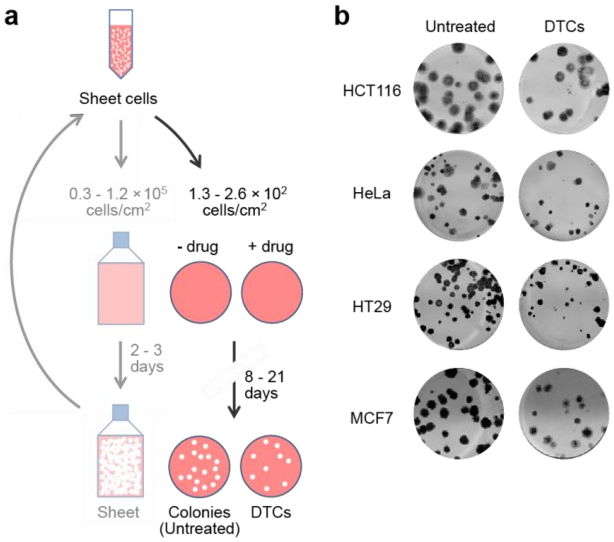

Figure S4. Generation of DTCs. (**a**) An illustration for generating sheet, untreated colonies and DTCs. (**b**) Untreated colonies and DTCs derived from HCT116, HeLa HT29 and MCF7 cells. DTCs were obtained from CIS at CoI_{50}.

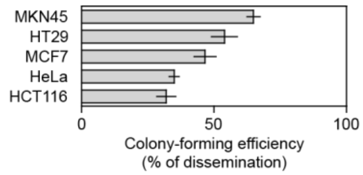

Figure S5. Colony formation efficiency of HCT116, HeLa, HT29, MCF7 and MKN45 cells. All experiments were performed in triplicate. Error bars represent s.e.m.

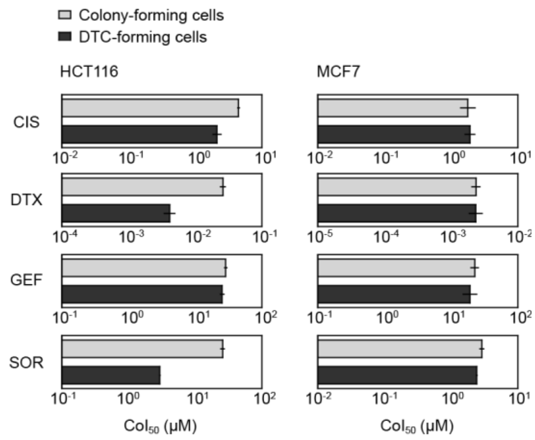

Figure S6. Drug sensitivities of untreated colony- and DTC-forming cells derived from HCT116 and MCF7 cells. CoI_{50} concentrations were determined based on dose-response curves for the number of colonies. Each experiment was performed in triplicate. Error bars represent s.e.m.

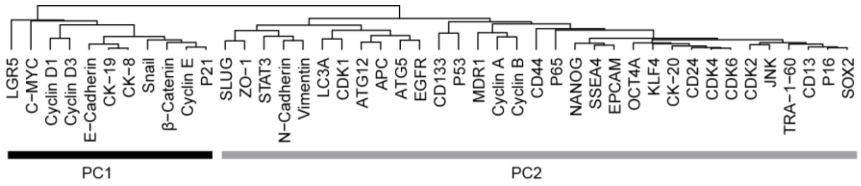

Figure S7. A detailed view of the dendrogram for protein axis in Fig. 3c.

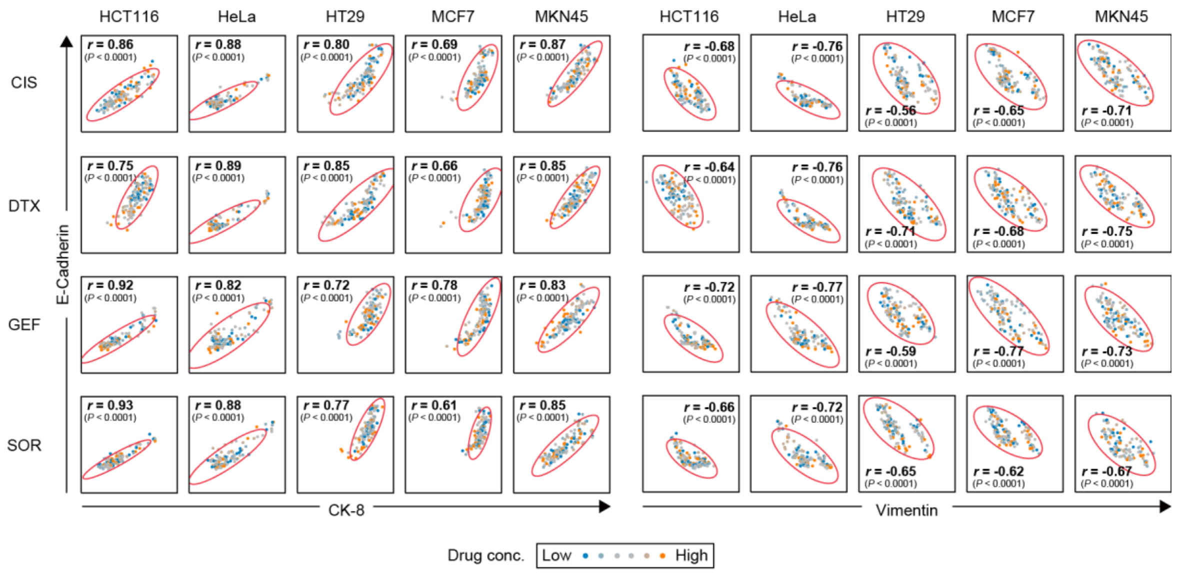

Figure S8. Validation of the integrity of the epithelial/non-epithelial relationship in the context of drug administration. Correlation of epithelial and mesenchymal markers in individual DTCs (*n* = 120 per panel). Scatter plots show the positive correlations between E-cadherin and CK-8 (left); and E-cadherin and vimentin show a negative correlation (right). Pearson's correlation coefficient (*r*) was used to assess these correlations. Each data point represents individual colonies from six different concentrations of each drug, as indicated by the colors in the legend.

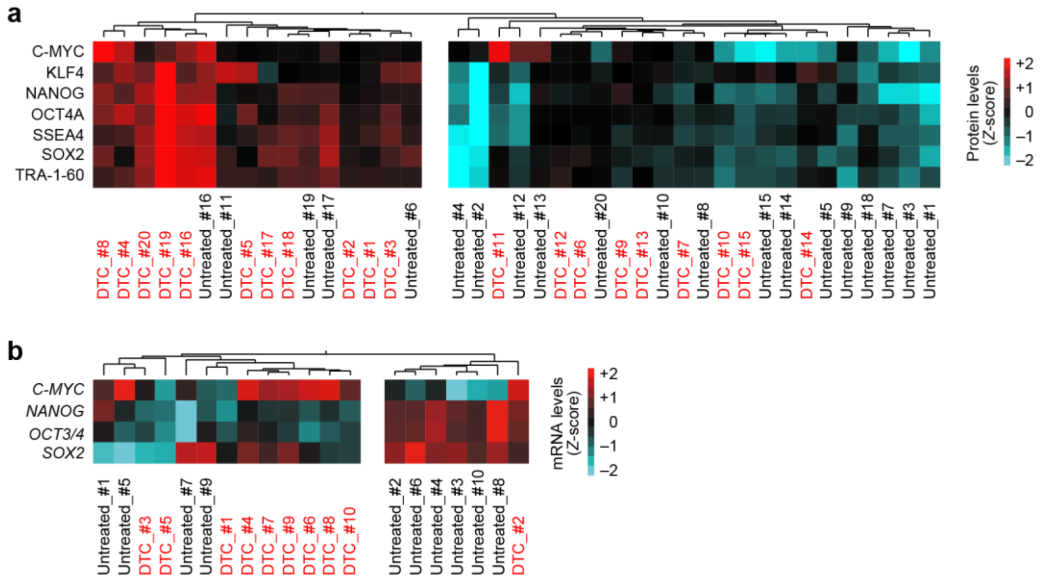

Figure S9. Comparison of protein and mRNA expression of stemness-associated proteins between untreated colonies and DTCs from MKN45. (a) Hierarchical clustering shows that two subtypes based on protein levels of indicated cell surface markers and pluripotent-inducing factors in untreated colonies and DTCs derived from MKN45 cells. Protein levels were from CoLA. DTCs emerged in the presence of 0.2 µM CIS. (b) Hierarchical clustering shows that two subtypes based on mRNA. mRNA levels were from colony qRT-PCR. DTCs emerged in the presence of 0.2 µM CIS.

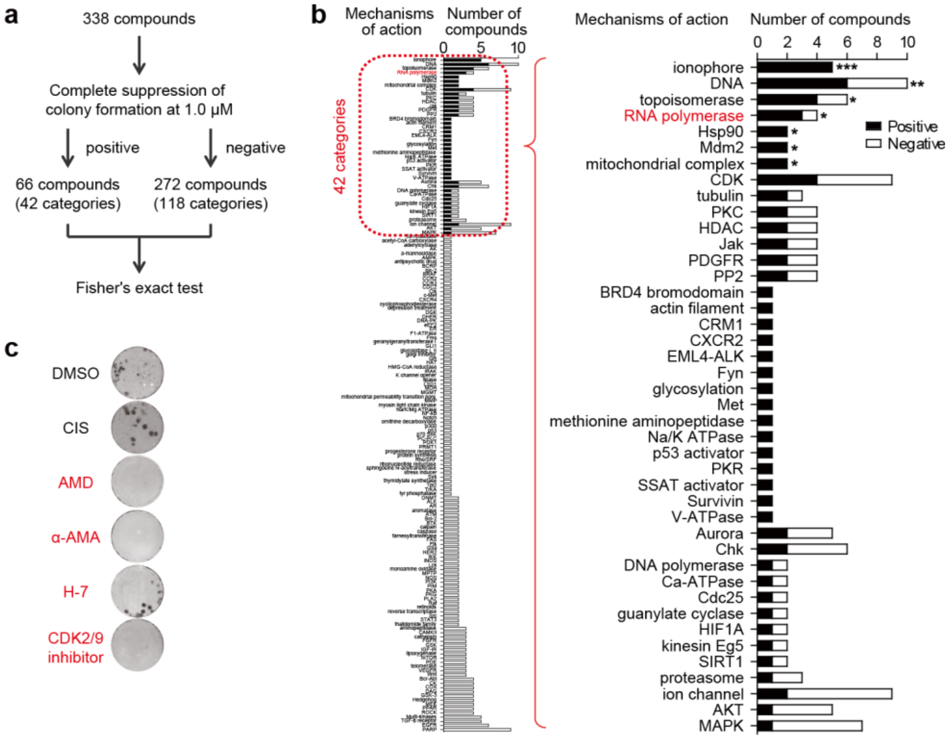

Figure S10. Significance of RNA polymerase inhibitors on colony formation assay-based compound screening. (**a**) A flow of colony formation assay-based compound screening. Sixty-six compounds that exhibited complete suppression of colony formation of MKN45 at 1.0 µM were identified from total 338 compounds. These compounds were sorted into 160 categories based on the target molecule or cellular biological process, and the categories were ranked based on *P*-values obtained with the use of Fisher's exact test. (**b**) Mechanism of action of positive compounds. *$P < 0.05$, **$P < 0.01$, **$P < 0.001$, Fisher's exact test. (**c**) Effect of transcription inhibitors on colony formation. Wells treated with 1.0 % (v/v) DMSO, 1.0 µM CIS, 1.0 µM AMD, 1.0 µM α-AMA, 1.0 µM H-7, and 1.0 µM CDK2/9 inhibitor were shown. AMD, α-AMA, H-7, and CDK2/9 inhibitor were categorized into RNA polymerase inhibitor. DMSO and CIS were showed as representative negative wells.

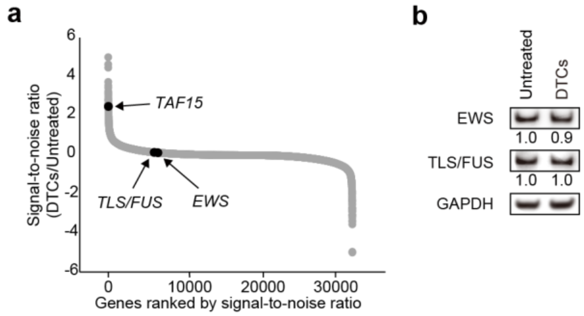

Figure S11. Gene and protein expression levels of TAF15, EWS, and TLS/FUS in DTCs. (**a**) Genes from the transcriptomic analysis in Fig. 6a are ranked according to their signal-to-noise ratio. *EWS*, *TLS/FUS*, *TAF15* were highlighted. (**b**) EWS and TLS/FUS protein levels in DTCs and untreated colonies derived from MKN45 cells by Western blot. The band intensities were relative to those of GAPDH. The numerical values are the relative intensity.

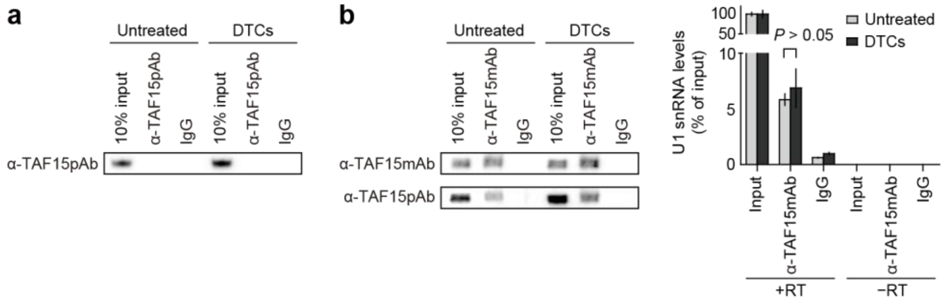

Figure S12. Immunoprecipitation of TAF15 and detection of TAF15-associated U1 snRNA from DTCs. (**a**) Validation of immunoprecipitation (IP) with an anti-TAF15 polyclonal antibody (α-TAF15pAb) by IP-Western blot. MKN45 DTCs were formed in the presence of 0.2 µM CIS. (**b**) Validation of IP with an anti-TAF15 monoclonal antibody (α-TAF15mAb) by IP-Western blot (left). qRT-PCR analysis of the TAF15-associated U1 snRNA levels in DTCs and untreated colonies (right). The U1 snRNA levels are relative to those of respective input. Each experiment was performed in three or four biological replicates. Error bars represent s.e.m. The one-tailed *P* values were obtained with Student's *t* test. +RT, with reverse transcription; –RT, without reverse transcription.

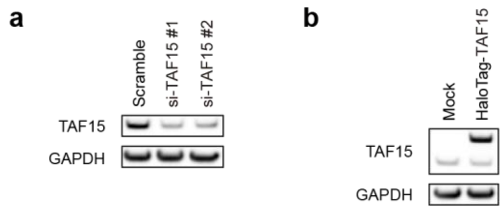

Figure S13. TAF15 knockdown and overexpression in MKN45. (**a**) TAF15 protein levels of MKN45 cells transfected with two independent TAF15-targeting siRNAs (si-TAF15) or a scramble siRNA. siRNAs correspond to Fig. 6g. (**d**) TAF15 protein levels of MKN45 cells that were transfected with the TAF15 expression vector (HaloTag-TAF15) or vehicle alone (Mock). The prominent higher molecular weight band indicates overexpressed HaloTag-TAF15 fusion protein. The faint bands indicate endogenous TAF15 in both Mock and HaloTag-TAF15.

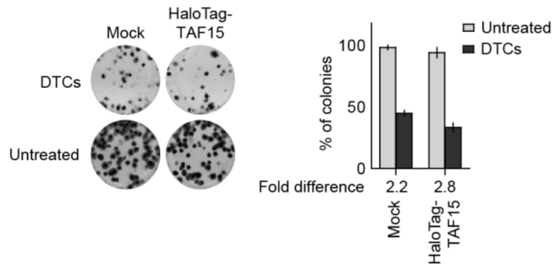

Figure S14. Effect of exogenous overexpression of TAF15 on DTC formation. MKN45 cells were transiently transfected with the TAF15 expression vector (HaloTag-TAF15) or vehicle alone (Mock). Cells were transfected prior to colony formation in the presence (DTCs) and absence (Untreated) of 0.2 µM CIS. The histogram shows the percent of colonies relative to untreated controls. Each experiment was performed in triplicate. Error bars represent s.e.m.

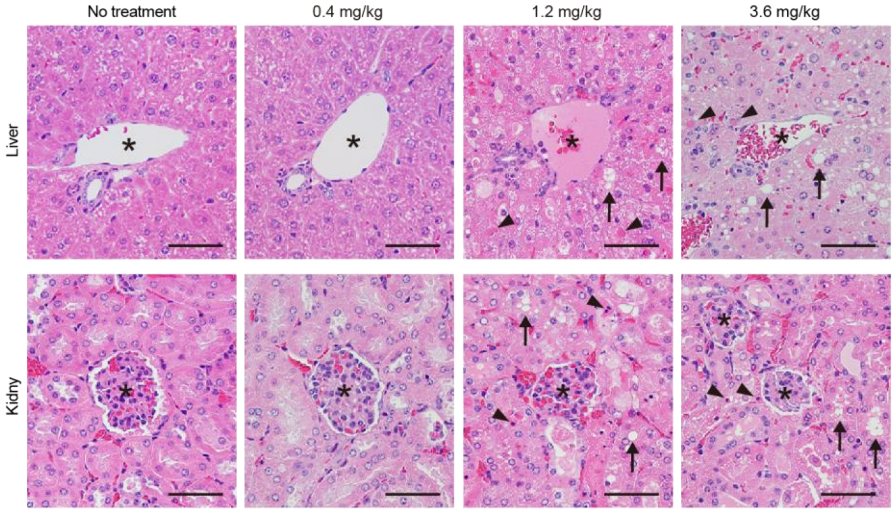

Figure S15. A minimum effect of α-AMA on cell and tissue morphology in liver and kidney at a dose of 0.4 mg/kg. Asterisks in the top and bottom panels indicate the portal vein and glomerulus, respectively. Arrows indicate representative vacuolated cytoplasm, and arrowheads indicate representative eosinophilic cytoplasm or condensed nuclei. Scale bars = 50 µm.

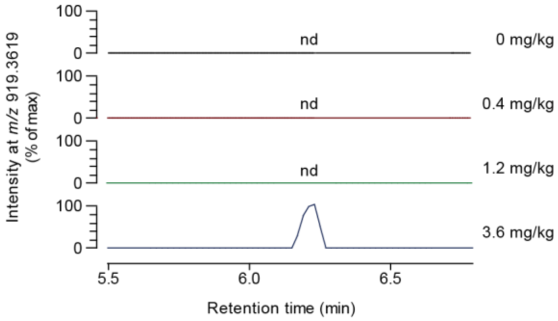

Figure S16. Detection limit of mass spectrometry on the analysis of α-AMA from the liver. The dose of α-AMA is shown on the right side of the corresponding data.

Supplementary Tables

Table S1. Initial drug concentrations for the six serial dilutions in Fig. 3a.

Drug	Initial concentration (μM)				
	HCT16	HeLa	HT29	MCF7	MKN45
CIS	0.2	2.0	0.2	2.0	0.2
DTX	1.2×10^{-4}	1.2×10^{-4}	1.2×10^{-4}	1.2×10^{-4}	1.2×10^{-4}
GEF	10.0	10.0	1.0	10.0	10.0
SOR	1.0	1.0	1.0	1.0	1.0

Table S2. Primary antibodies used in this study.

	Target	Vendor	Catalog no.	Dilution	Purpose
1	ATG12	Cell Signaling Technology	4180	1:100	CoLA
2	ATG5	Cell Signaling Technology	8540	1:100	CoLA
3	LCA3	Cell Signaling Technology	4599	1:100	CoLA
4	APC	Cell Signaling Technology	2504	1:100	CoLA
5	EGFR	Cell Signaling Technology	2232	1:100	CoLA
6	JNK	Becton Dickinson	610627	1:100	CoLA
7	P53	NeoMarkers	MS-187	1:200	CoLA
8	P65	Cell Signaling Technology	3034	1:100	CoLA
9	P16	Santa Cruz	sc-468	1:100	CoLA
10	P21	Cell Signaling Technology	2946	1:200	CoLA
11	CDK1	Becton Dickinson	610037	1:200	CoLA
12	CDK2	Becton Dickinson	610145	1:200	CoLA
13	CDK4	Becton Dickinson	610147	1:200	CoLA
14	CDK6	Cell Signaling Technology	3136	1:200	CoLA
15	Cyclin A	Becton Dickinson	611268	1:200	CoLA
16	Cyclin B	Becton Dickinson	610219	1:250	CoLA
17	Cyclin D1	Cell Signaling Technology	2926	1:250	CoLA
18	Cyclin D3	Cell Signaling Technology	2936	1:250	CoLA
19	Cyclin E	Cell Signaling Technology	4129	1:250	CoLA
20	β-Catenin	Cell Signaling Technology	9582	1:100	CoLA
21	CK-8	Thermo	MS-198	1:1000	CoLA
22	CK-19	Thermo	MS-377	1:1000	CoLA
23	CK-20	Thermo	MS-997	1:1000	CoLA
24	E-Cadherin	Cell Signaling Technology	3195	1:100	CoLA
25	ZO-1	Cell Signaling Technology	5406	1:100	CoLA
26	N-Cadherin	Cell Signaling Technology	4061	1:100	CoLA
27	SLUG	Cell Signaling Technology	9585	1:100	CoLA
28	Snail	Cell Signaling Technology	2879	1:100	CoLA
29	Vimentin	Cell Signaling Technology	5741	1:100	CoLA
30	CD13	Santa Cruz	sc-13536	1:100	CoLA
31	CD133	Cell Signaling Technology	3663	1:100	CoLA
32	CD24	Santa Cruz	sc-70598	1:100	CoLA
33	CD44	Cell Signaling Technology	3570	1:100	CoLA, FI
34	C-MYC	Santa Cruz	sc-40	1:100	CoLA
35	EPCAM	Santa Cruz	sc-71057	1:100	CoLA
36	KLF4	Cell Signaling Technology	4038	1:100	CoLA
37	LGR5	Becton Dickinson	562713	1:100	CoLA
38	MDR1	Sigma	P7965	1:100	CoLA
39	NANOG	Cell Signaling Technology	4903	1:100	CoLA
40	OCT4A	Cell Signaling Technology	2840	1:100	CoLA
41	SOX2	Cell Signaling Technology	3579	1:200	CoLA
42	SSEA4	Cell Signaling Technology	4755	1:200	CoLA
43	STAT3	Cell Signaling Technology	9132	1:100	CoLA
44	TRA-1-60	Cell Signaling Technology	4746	1:100	CoLA
45	5mC	Abcam	ab10805	1:300	MeDIP-chip
46	TAF15	Cell Signaling Technology	13150	1: 1000	WB
47	TAF15	provided by Dr. Tora	–	1:100	RIP
48	GAPDH	Cell Signaling Technology	2118	1:1000	WB
49	RNAPII	Cell Signaling Technology	2629	1:1000	WB
50	EWS	Cell Signaling Technology	11910	1:1000	WB

51	TLS/FUS	Cell Signaling Technology	4885	1:1000	WB
52	–	Cell Signaling Technology	5415	1:50	RIP

FI, fluorescent immunocytochemistry; 5mC, 5-methylcytosine; WB, Western blotting; RIP, RNA immunoprecipitation.

Table S3. Oligonucleotides used in this study.

	Gene	Accession	Forward (5′ to 3′)	Reverse (5′ to 3′)
1	*C-MYC*	NM_002467	CACACATCAGCACAACTACGC	CTCTTGACATTCTCCTCGGTG
2	*GAPDH*	NM_001256799	AATCCCATCACCATCTTCCA	TGGACTCCACGACGTACTCA
3	*NANOG*	NM_024865	TTGAAGCATCCGACTGTAAAG	CACTCTTCTCTGCAGAAGTGG
4	*OCT3/4*	NM_002701	GTGCCGTGAAGCTGGAGAAG	CTCAAAGCGGCAGATGGTC
5	*SOX2*	NM_003106	AATAAGTACTGGCGAACCATCTC	TAAATTACCAACGGTGTCAACC
6	*TAF15*	NM_003487	CCGACCATACTGATGACTGT	TTCACTACCCAAGAAGAGGC
